# *Starling*: Introducing a mesoscopic scale with *Confluence* for Graph Clustering

**DOI:** 10.1371/journal.pone.0290090

**Published:** 2023-08-24

**Authors:** Bruno Gaume

**Affiliations:** Centre National de la Recherche Scientifique, CLLE, ISCPIF, Toulouse, France; University of Delaware, UNITED STATES

## Abstract

Given a Graph *G* = (*V*, *E*) and two vertices *i*, *j* ∈ *V*, we introduce *Confluence*(*G*, *i*, *j*), a vertex mesoscopic closeness measure based on short Random walks, which brings together vertices from a same overconnected region of the Graph *G*, and separates vertices coming from two distinct overconnected regions. *Confluence* becomes a useful tool for defining a new Clustering quality function *Q*_*Conf*_(*G*, Γ) for a given Clustering Γ and for defining a new heuristic *Starling* to find a partitional Clustering of a Graph *G* intended to optimize the Clustering quality function *Q*_*Conf*_. We compare the accuracies of *Starling*, to the accuracies of three state of the art Graphs Clustering methods: *Spectral*-*Clustering*, *Louvain*, and *Infomap*. These comparisons are done, on the one hand with artificial Graphs (a) Random Graphs and (b) a classical Graphs Clustering *Benchmark*, and on the other hand with (c) *Terrain-Graphs* gathered from real data. We show that with (a), (b) and (c), *Starling* is always able to obtain equivalent or better accuracies than the three others methods. We show also that with the *Benchmark* (b), *Starling* is able to obtain equivalent accuracies and even sometimes better than an *Oracle* that would only know the expected overconnected regions from the *Benchmark*, ignoring the concretely constructed edges.

## 1 Introduction

*Terrain-Graphs* are real world Graphs that model data gathered by field work, in diverse fields such as sociology, linguistics, biology, or Graphs from the internet. Most *Terrain-Graphs* contrast with artificial Graphs (deterministic or Random) and share four similar properties [1–3]. They exhibit:

**p_1_**: Not many edges : *m* being *O*(*n*.log(*n*)) (where *m* is the number of edges and *n* the number of vertices);
**p_2_**: Short paths (*L*, the average number of edges on the shortest path between two vertices is low);
**p_3_**: A high Clustering rate $C\;=\;\frac{3\;\times \;number\;of\;triangles}{number\;of\;connected\;triplets}$ (many overconnected local subGraphs in a globally sparse Graph);
**p_4_**: A heavy-tailed degree distribution (the distribution of the degrees of the vertices of the Graph can be approximated by a power law).

Clustering a *Terrain*-*Graph* consists of grouping together in Modules vertices that belong to the same overconnected region of the Graph (property *p*_3_), while keeping separate vertices that do not (property *p*_1_). These groups of overconnected vertices form an essential feature of the structures of most *Terrain-Graphs*. Their detection is central in a wide variety of fields, such as in biology [4], in sociology [5], in linguistics [6] or in computer sciences [7], for many tasks as the grouping of most diverse entities [8–13], the pattern detection in data [14], the prediction of links [15], the model training [16], the label assignment [17], the recommender Algorithms [18], the data noise removal [19], or the feature matching [20].

In section 2 we put in context in the state of the art, the methods with which we compare our results: in section 2.1 we present the *Spectral*-*Clustering*, one of the most popular and efficient Graph Clustering methods, in section 2.2.1 *Louvain*, one of the most used Graph Clustering method optimizing *Modularity* the most popular Graph Clustering quality function, and in section 2.2.2 *Infomap*, one of the most efficient Graph Clustering method optimizing the most elegant Graph Clustering quality function.

In section 3 we present the *Confluence*, a vertex mesoscopic closeness measure and a new Clustering quality function *Q*_*Conf*_ based on the *Confluence*. In section 4 we compare optimality for *Modulatity* and optimality for *Q*_*Conf*_. In section 5 we propose to consider a clustering method, as Binary Edge-Classifier By nodes Blocks (*BECBB*) trying to classify each pairs of vertices into two classes: the edges and the non-edges. In section 6 we propose a heuristic *Starling* for optimizing the objective function *Q*_*Conf*_.

In section 7, we compare the accuracies as *BECBB*, of *Starling*, *Louvain*, *Infomap* and *Spectral*-*Clustering*. These comparisons are done, on the one hand with artificial Graphs (a) Random Graphs and (b) a classical Graphs Clustering *Benchmark*, and on the other hand with (c) *Terrain-Graphs* gathered from real data. We show that with (a), (b) and (c), *Starling* is always able to obtain equivalent or better accuracies than the three others methods. We show also that with the *Benchmark* (b), *Starling* is able to obtain equivalent accuracies and even sometimes better than an *Oracle* that would only know the expected overconnected regions from the *Benchmark*, ignoring the concretely constructed edges that are to be predicted by the *Oracle* as *BECBB*.

In section 8 we discuss the choice of parameters, and conclude in section 9.

## 2 Previous work

The literature on Graph Clustering is too extensive for a comprehensive review here. We concentrate on placing in the state of art, the methods to which we compare our results.

**Let G = (V, E) be a Graph with n = |V| vertices and m = |E| edges**.

$P_{2}^{V}:P_{2}^{V}=\{X\subseteq V\;\text{such}\;|X|=2\}$ ;
**Degree**: The degree of a vertex *i* in *G* is *d*_*G*_(*i*) = |{*j* ∈ *V*/{*i*, *j*} ∈ *E*}|;
**Module**: A *Module* *γ* of *G* is a non-empty subset of the Graph’s vertices: *γ* ≠ ⌀ and *γ* ⊆ *V*;
**Clustering**: A *Clustering* Γ of *G* is a set of Modules of *G* such that ⋃_*γ*∈Γ_ *γ* = *V*;
**Partitional Clustering**: If $\forall \gamma_{i},\gamma_{j}\in \Gamma ,\;\left(i\neq j\right)\;\Rightarrow \;\left(\gamma_{i}\cap \gamma_{j}=⌀\right)$, then Γ is a *Partitional Clustering* of *G*, where Modules of *G* are not allowed to overlap. Given such a Γ we can define an equivalence relation $\overset{\Gamma }{∼}$ on the set of vertices: $\forall u,v\in V,\;\left(u\;\overset{\Gamma }{∼}\;v\right)\Leftrightarrow \left(\exists \gamma \in \Gamma \;\text{such}\;\text{that}\;u\in \gamma \;\text{and}\;v\in \gamma \right)$.

### 2.1 Spectral Graph Clustering

Spectral Graph Clustering is one of the most popular and efficient Graph Clustering Algorithms. It generally use the classical *kmeans* Algorithm whose original idea was proposed by Hugo Steinhaus [21]. Spectral Graph Clustering Algorithms work as follows (see [22]):

**Algorithm 1** *SGC*: Spectral Graph Clustering

**Input**:

 *G* = (*V*, *E*) an undirected Graph with |*V*| = *n*

κ∈N such 0 < *κ* ≤ *n* (*κ* is the desired number of Modules).

**Output**: A Partitional Clustering of *G* with *κ* Modules

** (1)** Form the Adjacency Matrix $A={\left(a_{i,j}\right)}_{i,j\in V}$ with $a_{i,j}=\{\begin{array}{l} 1\;\text{if}\;\{i,j\}\in E,\\ 0\;\text{otherwise}. \end{array}$

** (2)** Form the Degree Matrix $D={\left(d_{i,j}\right)}_{i,j\in V}$ with $d_{i,j}=\{\begin{array}{l} d_{G}(i)\;\text{if}\;\{i=j\},\\ 0\;\text{otherwise}. \end{array}$

** (3)** Let $L\in R^{n\times n}$ the *Normalized Graph Laplacian*: *L* = *I* − *D*^−1^*A* (where *I* is the identity matrix $\in R^{n\times n}$).

** (4)** Compute the first *κ* eigenvectors *u*_1_, …, *u*_*k*_ of *L* (see [23]).

** (5)** Let $U\in R^{n\times \kappa }$ be the matrix containing the vectors *u*_1_, …, *u*_*κ*_ as columns.

** (6)** For *i* = 1, …, *n*, let $y_{i}\in R^{\kappa }$ be the vector corresponding to the *i*-th row of *U*.

** (7)** Cluster the points ${\left(y_{i}\right)}_{i=1,\ldots ,n}\in R^{\kappa }$ with the *k*-*means* Algorithm into *κ* clusters *C*_1_, …, *C*_*k*_.

** (8)** For *i* = 1, …, *κ*, let $c_{i}=\left\{j|y_{j}\in C_{i}\right\}$.

**Return** {*c*_1_, …, *c*_*κ*_}

We can notice that for Spectral Graph Clustering in Algorithm 1, we need to know *κ* the number of groups of vertices in advance in the *Input*. It is an advantage because it makes it possible to have a handle on the desired number of Modules, but how to choose *κ* when one does not know the structure of the Graph? The choice of the number *κ* of groups is fundamental, it is not a simple problem (see [23–31]), and the quality of the results varies greatly depending on *κ*, what we confirm in section 7.2.1 with Figs 7 and 8.

### 2.2 When we don’t know the number of groups in advance

**Let G = (V, E) be a Graph and Γ a Partitional Clustering of its vertices**.

**Clustering quality function**: A Clustering quality function *Q*(*G*, Γ) is an R-valued function designed to measure the adequacy of the Modules with the overconnected regions of *Terrain-Graphs* (property *p*_3_).

When we don’t know κ the number of groups of vertices in advance, given a Clustering quality function *Q*, in order to establish a good Partitional Clustering for a Graph *G* = (*V*, *E*), it would be sufficient to build all the possible partitionings of the set of vertices *V*, and to pick a partitioning Γ such that *Q*(*G*, Γ) is optimal. This method is however obviously concretely impractical, since the number of partionings of a set of size *n* = |*V*| is equal to the *n*^*th*^ Bell number, a sequence known to grow exponentially [32]. Many Graph Clustering methods therefore consist in defining a heuristic that can find in a reasonable amount of time a Clustering Γ that tentatively optimises *Q*(*G*, Γ) for a given Clustering quality function *Q*.

With methods optimizing a quality function *Q*, we do not need to know *κ* the number of vertices groups in advance in the input, because *κ* is then a direct consequence of the quality function *Q*: *κ* will be automatically built by the optimisation of *Q*.

#### 2.2.1 Louvain

The **Louvain** method proposed in 2008 by Blondel, Guillaume, Lambiotte, and Lefebvre in [33] is a heuristic for tentatively maximizing the quality function *Modularity* proposed in 2004 by Newman and Girvan [34]. The modularity of a Partitional Clustering for a Graph *G* = (*V*, *E*) with *m* = |*E*| edges is equal to the difference between the proportion of links internal to Modules of the Clustering, and the same quantity expected in a null model, where no community structure is expected. The null model is a Random Graph *G*_*Null*_ with the same number of vertices and edges, as well as the same distribution of degrees as *G*, where the probability of having an edge between two vertices *x* and *y* is equal to $\frac{d_{G}\left(x\right).d_{G}\left(y\right)}{2m}$.

Let *G* = (*V*, *E*) be a Graph with *m* edges and Γ a partitioning of *V*. The modularity of Γ can be defined as follows. The definition of modularity given by Newman and Girvan in [34], is equivalent to that we propose here in Formula 1:

$$\begin{array}{c} Modularity\left(G,\Gamma \right)=\frac{1}{2m}\;\sum_{\gamma \in \Gamma }\;\sum_{i,j\in \gamma }\;P_{edge}\left(G,i,j\right)-P_{edge}\left(G_{Null},i,j\right) \end{array}$$ (1)

Where *P*_*edge*_(*G*, *x*, *y*) is a symmetrical vertex closeness measure equal to the probability of {*x*, *y*} being an edge of *G*, that is:

$$\begin{array}{c} P_{edge}\left(G,i,j\right)=\{\begin{array}{c} 1\;\text{if}\;\{i,j\}\in E,\\ 0\;\text{otherwise}. \end{array} \end{array}$$ (2)

$$\begin{array}{c} P_{edge}\left(G_{Null},i,j\right)=\frac{d_{G}\left(i\right).d_{G}\left(j\right)}{2m} \end{array}$$ (3)

In Eq 1, the first term $\frac{1}{2m}$ is purely conventional, so that the modularity values all live in the [−1, 1] interval, but plays no role when maximizing modularity, since it is constant for a given Graph *G*.

We then define $Q_{P_{edge}}$ as Newman and Girvan’s quality function, to be maximized:

$$\begin{array}{c} Q_{P_{edge}}\left(G,\Gamma \right)=\sum_{\gamma \in \Gamma }\;\sum_{i,j\in \gamma }\;P_{edge}\left(G,i,j\right)-P_{edge}\left(G_{Null},i,j\right) \end{array}$$ (4)

$$\begin{array}{c} =\sum_{\gamma \in \Gamma }\;\sum_{i,j\in \gamma }\;\{\begin{array}{c} 1-\frac{d_{G}\left(i\right).d_{G}\left(j\right)}{2m}\;\text{if}\;\left\{i,j\right\}\in E,\\ -\frac{d_{G}\left(i\right).d_{G}\left(j\right)}{2m}\;\text{otherwise}. \end{array} \end{array}$$ (5)

**For Louvain, a good Partitional Clustering Γ** as per 5 is one that groups in the same Module vertices that are linked (especially ones with low degrees, but also to a lesser extent ones with high degrees), while avoiding as much as possible the grouping of non-linked vertices (especially ones with high degrees, but to a lesser extent ones with low degrees).

However, several authors [35, 36] showed that optimizing *Modularity* leads to merging small Modules into larger ones, even when those small Modules are well defined and weakly connected to one another. To address this problem, some authors [37, 38] defined multiresolution variants of *Modularity*, adding a resolution parameter to control the size of the Modules.

For instance [37] introduces a parameter λ∈R in Eq 5:

$$\begin{array}{c} Q_{\lambda }=\sum_{\gamma \in \Gamma }\;\sum_{i,j\in \gamma }\;\{\begin{array}{c} 1-\lambda .\frac{d_{G}\left(i\right).d_{G}\left(j\right)}{2m}\;\text{if}\;\left\{i,j\right\}\in E,\\ -\lambda .\frac{d_{G}\left(i\right).d_{G}\left(j\right)}{2m}\;\text{otherwise}. \end{array} \end{array}$$ (6)

where λ is a resolution parameter: the higher the resolution λ, the smaller the Modules get.

Nevertheless, in [39], the authors show that *“… multiresolution Modularity suffers from two opposite coexisting problems: the tendency to merge small subGraphs, which dominates when the resolution is low; the tendency to split large subGraphs, which dominates when the resolution is high. In benchmark networks with heterogeneous distributions of cluster sizes, the simultaneous elimination of both biases is not possible and multiresolution Modularity is not capable to recover the planted community structure, not even when it is pronounced and easily detectable by other methods, for any value of the resolution parameter. This holds for other multiresolution techniques and it is likely to be a general problem of methods based on global optimization*.

*[…] real networks are characterized by the coexistence of clusters of very different sizes, whose distributions are quite well described by power laws* [40, 41]. *Therefore there is no characteristic cluster size and tuning a resolution parameter may not help.”*

The *Louvain* method https://github.com/10XGenomics/louvain is non-deterministic, i.e. each time *Louvain* is run on the same Graph, the results may vary slightly. In the rest of this paper all the results concerning the *Louvain* method on a given Graph are the result of a single run on this Graph.

#### 2.2.2 Infomap

The **Infomap** method is a heuristic for tentatively maximizing the quality function described in 2008 by Rosvall and Bergstrom [42]. This quality function is based on the minimum description length principle [43]. It consists in measuring the compression ratio that a given partitioning Γ provides for describing the trajectory of a Random walk on a Graph. The trajectory description happens on two levels. When the walker enters a Module, we write down its name. We then write the vertices that the walker visits, with a notation local to the Module, so that an identical short name may be used for different vertices from different Modules. A concise description of the trajectory, with a good compression ratio, is therefore possible when the Modules of Γ are such that the walker tends to stay in them, which corresponds to the idea that the walker is trapped when it enters a good Module, which is supposed to be a overconnected region that is only weakly connected to other Modules.

**For Infomap, a good Partitional Clustering Γ** is then one that groups in same Module vertices allowing a good compression ratio for describing the trajectory of a Random walker on *G*.

However, as we will see in section 7, *Infomap* only identifies a single Module when the overconnected regions are only sligthly pronounced.

The *Infomap* method https://github.com/mapequation/ is non-deterministic, in the rest of this paper all the results concerning the *Infomap* method on a given Graph are the result of a single run on this Graph.

## 3 *Confluence*, a vertices mesoscopic closeness measure

The definition of *Confluence* proposed in this section is an adaptation of these proposed in [44] to compare the structures of two *Terrain-Graphs*.

In Eq 5, with regards to a Graph *G*:

-
$P_{edge}\left(G,i,j\right)=\{\begin{array}{c} 1\;\;\text{if}\;\;\{i,j\}\in E,\\ 0\;\text{otherwise}. \end{array}$ is a **local** (microscopic) vertices closeness measure relative to *G*;
-
$P_{edge}\left(G_{Null},i,j\right)=\frac{d_{G}\left(i\right).d_{G}\left(j\right)}{2m}$ is a **global** (macroscopic) vertices closeness measure relative to *G*.

To avoid the resolution limits of *Modularity* described in [35–39], we introduce here *Confluence*(*G*, *i*, *j*), an intermediate **mesoscopic** vertices closeness measure relative to a Graph *G*, that we define below.

If *G* = (*V*, *E*) is a reflexive and undirected Graph, let us imagine a walker wandering on the Graph *G*: at time t∈N, the walker is on one vertex *i* ∈ *V*; at time *t* + 1, the walker can reach any neighbouring vertex of *i*, with a uniform probability. This process is called a simple Random walk [45]. It can be defined by a Markov chain on *V* with an *n* × *n* transition Matrix [*G*]:

$$\begin{array}{c} \left[G\right]={\left(g_{i,j}\right)}_{i,j\in V}\;\;\text{with}\;\;g_{i,j}=\{\begin{array}{c} \frac{1}{d_{G}\left(i\right)}\;\text{if}\;\left\{i,j\right\}\in E\text{,}\\ 0\;\text{otherwise}. \end{array} \end{array}$$ (7)

Since *G* is reflexive, each vertex has at least one neighbour (itself) and [*G*] is therefore well defined. Furthermore, by construction, [*G*] is a stochastic Matrix: ∀*i* ∈ *V*, ∑_*j*∈*V*_ *g*_*i*,*j*_ = 1. The probability $P_{G}^{t}\left(i⇝j\right)$ of a walker starting on vertex *i* and reaching vertex *j* after *t* steps is:

$$\begin{array}{c} P_{G}^{t}\left(i⇝j\right)={\left({\left[G\right]}^{t}\right)}_{i,j} \end{array}$$ (8)

**Proposition 1** *Let G* = (*V*, *E*) *be a reflexive Graph with m edges, and G*_*null*_ = (*V*, *E*_*null*_) *its null model such that the probability of the existence of a link between two vertices i and j is* $e_{i,j}=\frac{d_{G}\left(i\right).d_{G}\left(j\right)}{2m}$.

$$\begin{array}{c} \forall t\in N^{*},\;\forall i,j\in V,\;P_{G_{null}}^{t}\left(i⇝j\right)=\frac{d_{G}\left(j\right)}{2m} \end{array}$$ (9)

**Proof** by induction on *t*:

**(a) True for t = 1**:

$$\begin{array}{c} \begin{array}{c} \forall i,j\in V,P_{G_{null}}^{1}\left(i⇝j\right)=e_{i,j}.\frac{1}{d_{G}\left(i\right)}=\frac{d_{G}\left(i\right).d_{G}\left(j\right)}{2m}.\frac{1}{d_{G}\left(i\right)}=\frac{d_{G}\left(j\right)}{2m} \end{array} \end{array}$$

**(b) If true for t then true for t + 1**:

$$\begin{array}{c} \begin{array}{c} \forall i,j\in V,P_{G_{null}}^{t+1}\left(i⇝j\right)=\sum_{k\in V}(P_{G_{null}}^{t}\left(i⇝k\right).P_{G_{null}}^{1}\left(k⇝j\right))\\ =\sum_{k\in V}(P_{G_{null}}^{t}\left(i⇝k\right).\frac{d_{G}\left(j\right)}{2m})=\frac{d_{G}\left(j\right)}{2m}.\sum_{k\in V}P_{G_{null}}^{t}\left(i⇝k\right)\\ =\frac{d_{G}\left(j\right)}{2m}.\sum_{k\in V}\frac{d_{G}\left(k\right)}{2m}=\frac{d_{G}\left(j\right)}{2m} \end{array} \end{array}$$

**(a) & (b) ⇒ 9**

**On a Graph G = (V, E)** the trajectory of a Random walker is completely governed by the topology of the Graph in the vicinity of the starting node: after *t* steps, any vertex *j* located at a distance of *t* links or less can be reached. The probability of this event depends on the number of paths between *i* and *j*, and on the structure of the Graph around the intermediary vertices along those paths. The more short paths exist between vertices *i* and *j*, the higher the probability $P_{G}^{t}\left(i⇝j\right)$ of reaching *j* from *i*.
**On the Graph G_null_** the trajectory of a Random walker is only governed by the degrees of the vertices *i* and *j*, and no longer by the topology of the Graph in the vicinity of these to nodes.

We want to consider as “close” each pair of vertices {*i*, *j*} having a probability of reaching *j* from *i* after a short Random walk in *G*, greater than the probability of reaching *j* from *i* in *G*_*null*_. We therefore define the *t*-confluence *Conf*^*t*^(*G*, *i*, *j*) between two vertices *i*, *j* on a Graph *G* as follows:

$$\begin{array}{c} Conf^{t}\left(G,i,j\right)=\{\begin{array}{c} 0\;\text{if}\;i=j,\\ \frac{P_{G}^{t}\left(i⇝j\right)-P_{G_{null}}^{t}\left(i⇝j\right)}{P_{G}^{t}\left(i⇝j\right)+P_{G_{null}}^{t}\left(i⇝j\right)}=\frac{P_{G}^{t}\left(i⇝j\right)-\frac{d_{G}\left(j\right)}{2m}}{P_{G}^{t}\left(i⇝j\right)+\frac{d_{G}\left(j\right)}{2m}}\;\text{otherwise}. \end{array} \end{array}$$ (10)

**Proposition 2** *Let G* = (*V*, *E*) *be a reflexive Graph with m edges, and G*_*null*_ *its null model such that the probability of the existence of a link between two vertices i and j is* $e_{i,j}=\frac{d_{G}\left(i\right).d_{G}\left(j\right)}{2m}$.

$$\begin{array}{c} \forall t\in N^{*},\;\forall i,j\in V,\;Conf^{t}\left(G_{Null},i,j\right)=0 \end{array}$$ (11)

**Proof**:

$$\begin{array}{c} \text{If}\;i=j,\;\text{the}\;\text{result}\;\text{follows}\;\text{directly}\;\text{from}\;\text{definition}\;10.\\ \text{If}\;i\neq j,{Conf}^{t}(G_{Null},i,j)=\frac{P_{G_{Null}}^{t}\left(i⇝j\right)-\frac{d_{G_{Null}}\left(j\right)}{2m}}{P_{G_{Null}}^{t}\left(i⇝j\right)+\frac{d_{G_{Null}}\left(j\right)}{2m}}\;(\text{by}\;\text{definition}\;10)\\ =\frac{P_{G_{Null}}^{t}\left(i⇝j\right)-\frac{d_{G}\left(j\right)}{2m}}{P_{G_{Null}}^{t}\left(i⇝j\right)+\frac{d_{G}\left(j\right)}{2m}}\;(\text{by}\;\text{definition}\;\text{of}\;G_{Null})\\ =\frac{\frac{d_{G}\left(j\right)}{2m}-\frac{d_{G}\left(j\right)}{2m}}{\frac{d_{G}\left(j\right)}{2m}+\frac{d_{G}\left(j\right)}{2m}}\;(\text{by}\;\text{proposition}\;1)\\ =0 \end{array}$$

To prove that *Conf*^*t*^(*G*, ⋅, ⋅) is symmetric, we first need to prove proposition 3.

**Proposition 3** *Let G* = (*V*, *E*) *be a reflexive Graph*.

$$\begin{array}{c} \forall t\in N^{*},\;\forall i,j\in V,P_{G}^{t}\left(i⇝j\right)=\frac{d_{G}\left(j\right)}{d_{G}\left(i\right)}.P_{G}^{t}\left(j⇝i\right) \end{array}$$ (12)

**Proof** by induction on *t*:

**(a) True for t = 1**:

$$\begin{array}{c} \forall i,j\in V,\text{if}\;\left\{i,j\right\}\notin E,\;\text{then}\;P_{G}^{1}\left(i⇝j\right)=0\;\text{and}\;P_{G}^{1}\left(j⇝i\right)=0,\\ \text{therefore}\;P_{G}^{1}\left(i⇝j\right)=\frac{d_{G}\left(j\right)}{d_{G}\left(i\right)}.P_{G}^{1}\left(j⇝i\right)=0\\ \text{otherwise}\;P_{G}^{1}\left(i⇝j\right)=\frac{1}{d_{G}\left(i\right)}=\frac{d_{G}\left(j\right)}{d_{G}\left(i\right)}.\frac{1}{d_{G}\left(j\right)}=\frac{d_{G}\left(j\right)}{d_{G}\left(i\right)}.P_{G}^{1}\left(j⇝i\right) \end{array}$$

**(b) If true for t then true for t + 1**:

$$\begin{array}{c} \forall i,j\in V,P_{G}^{t+1}\left(i⇝j\right)=\sum_{k\in V}(P_{G}^{t}\left(i⇝k\right).P_{G}^{1}\left(k⇝j\right))\\ =\sum_{k\in V}(P_{G}^{t}\left(k⇝i\right).\frac{d_{G}\left(k\right)}{d_{G}\left(i\right)}.P_{G}^{1}\left(k⇝j\right))=\sum_{k\in V}(P_{G}^{t}\left(k⇝i\right).\frac{d_{G}\left(k\right)}{d_{G}\left(i\right)}.P_{G}^{1}\left(j⇝k\right).\frac{d_{G}\left(j\right)}{d_{G}\left(k\right)})\\ =\sum_{k\in V}(P_{G}^{t}\left(k⇝i\right).P_{G}^{1}\left(j⇝k\right).\frac{d_{G}\left(j\right)}{d_{G}\left(i\right)})=\frac{d_{G}\left(j\right)}{d_{G}\left(i\right)}\sum_{k\in V}(P_{G}^{1}\left(j⇝k\right).P_{G}^{t}\left(k⇝i\right))\\ =\frac{d_{G}\left(j\right)}{d_{G}\left(i\right)}.P_{G}^{t+1}\left(j⇝i\right) \end{array}$$

**(a) & (b) ⇒ 12**

**Proposition 4** *Let G* = (*V*, *E*) *be a reflexive Graph*.

$$\begin{array}{c} \forall t\in N^{*},\;\forall i,j\in V,Conf^{t}\left(G,i,j\right)=Conf^{t}\left(G,j,i\right) \end{array}$$ (13)

**Proof**:

$$\begin{array}{c} \text{If}\;\text{i = j}\;\text{:}\;\text{it}\;\text{follows}\;\text{directly}\;\text{from}\;\text{definition}\;\text{10}.\\ \text{If}\;i\neq j:Conf^{t}\left(G,i,j\right)=\frac{P_{G}^{t}\left(i⇝j\right)-P_{G_{null}}^{t}\left(i⇝j\right)}{P_{G}^{t}\left(i⇝j\right)+P_{G_{null}}^{t}\left(i⇝j\right)}=\frac{P_{G}^{t}\left(i⇝j\right)-\frac{d_{G}\left(j\right)}{2m}}{P_{G}^{t}\left(i⇝j\right)+\frac{d_{G}\left(j\right)}{2m}}=\frac{\frac{d_{G}\left(j\right)}{d_{G}\left(i\right)}.P_{G}^{t}\left(j⇝i\right)-\frac{d_{G}\left(j\right)}{2m}}{\frac{d_{G}\left(j\right)}{d_{G}\left(i\right)}.P_{G}^{t}\left(j⇝i\right)+\frac{d_{G}\left(j\right)}{2m}}\\ =\frac{\left(\frac{d_{G}\left(j\right)}{d_{G}\left(i\right)}.P_{G}^{t}\left(j⇝i\right)-\frac{d_{G}\left(j\right)}{2m}\right).\frac{d_{G}\left(i\right)}{d_{G}\left(j\right)}}{\left(\frac{d_{G}\left(j\right)}{d_{G}\left(i\right)}.P_{G}^{t}\left(j⇝i\right)+\frac{d_{G}\left(j\right)}{2m}\right).\frac{d_{G}\left(i\right)}{d_{G}\left(j\right)}}=\frac{P_{G}^{t}\left(j⇝i\right)-\frac{d_{G}\left(i\right)}{2m}}{P_{G}^{t}\left(j⇝i\right)+\frac{d_{G}\left(i\right)}{2m}}=\frac{P_{G}^{t}\left(j⇝i\right)-P_{G_{null}}^{t}\left(j⇝i\right)}{P_{G}^{t}\left(j⇝i\right)+P_{G_{null}}^{t}\left(j⇝i\right)}\\ =Conf^{t}\left(G,j,i\right) \end{array}$$

Most *Terrain-Graphs* exhibit the properties *p*_2_ (short paths) and *p*_3_ (high Clustering rate). With a classic distance such as *the shortest path between two vertices*, all vertices would be close to each other in a *Terrain*-*Graph* (because of property *p*_2_). On the contrary, *Confluence* allows us to identify vertices living in a same overconnected region of *G* (property *p*_3_):

If *i*, *j* are in a same overconnected region:
  $$\begin{array}{c} \;P_{G}^{t}\left(i⇝j\right)>P_{G_{null}}^{t}\left(i⇝j\right)\text{,}\;\text{thus}\;Conf\left(G,i,j\right)>0\;\;\;\;\; \end{array}$$ (14)
If *i*, *j* are in two distinct overconnected regions:
  $$\begin{array}{c} P_{G}^{t}\left(i⇝j\right)<P_{G_{null}}^{t}\left(i⇝j\right)\text{,}\;\text{thus}\;Conf\left(G,i,j\right)<0 \end{array}$$ (15)
  Where the notion of *region* varies according to t:

- **When t = 1**: $Conf^{t}\left(G,i,j\right)=\{\begin{array}{c} \frac{2m-d_{G}\left(i\right).d_{G}\left(j\right)}{2m+d_{G}\left(i\right).d_{G}\left(j\right)}\;\text{if}\;\left\{i,j\right\}\in E,\\ -1\;\text{otherwise}. \end{array}$ *Confluence* is a **microscopic** vertices closeness measure relative to *G*. The notion of **region in this case has a radius = 1**, it is the notion of *neighborhood*. *Confluence* is then **independent of the intermediate structures** between the two vertices *i* and *j* in *G*;
- **When 1 < t < ∞**: $Conf^{t}\left(G,i,j\right)=\frac{P_{G}^{t}\left(i⇝j\right)-\frac{d_{G}\left(j\right)}{2m}}{P_{G}^{t}\left(i⇝j\right)+\frac{d_{G}\left(j\right)}{2m}}$, *Confluence* is a **mesoscopic** vertices closeness measure relative to *G*. The notion of **region in this case has a 1 < radius = t < ∞**, it is no longer a local notion as the notion of *neighborhood*. *Confluence* is then **sensitive to the t-intermediate structures (t-mesoscopicity)** between the two vertices *i* and *j* in *G* (see 14 and 15);
- **When t → ∞**: lim_*t*→∞_ *Conf*^*t*^(*G*, *i*, *j*) = 0, and *Confluence* is no longer sensitive to any structure in *G*. (lim_*t*→∞_ *Conf*^*t*^(*G*, *i*, *j*) = 0 because we can prove with the Perron-Frobenius theorem [46] that if *G* is reflexive and strongly connected, then the Matrix [*G*] is ergodic [47], then ${\text{lim}}_{t\to \infty }\;P_{G}^{t}\left(i⇝j\right)=\frac{d_{G}\left(j\right)}{2m}$. So by definition 10 and proposition 1: lim_*t*→∞_ *Conf*^*t*^(*G*, *i*, *j*) = 0).

*Confluence* actually defines an infinity of mesoscopic vertex closeness measures, one for each Random walk of length 1 < *t* < ∞. For clarity, in the rest of this paper, we set *t* = 3 and define *Conf*(*G*, *i*, *j*) = *Conf*^3^(*G*, *i*, *j*).

### 3.1 Using a mesoscopic scale with *Confluence* for a new Clustering quality function

We propose here $Q_{Conf}^{\tau }$, a new Clustering quality function, which introduces a mesoscopic scale through *Confluence* with a resolution parameter *τ* ∈ [0, 1] to promote density of the Modules:

$$\begin{array}{c} Q_{1}\left(G,\Gamma \right)=\sum_{\gamma \in \Gamma }\;\sum_{i\neq j\in \gamma }\{\begin{array}{c} +1-\frac{d_{G}\left(i\right).d_{G}\left(j\right)}{2m}\;\text{if}\;\left\{i,j\right\}\in E,\\ -1-\frac{d_{G}\left(i\right).d_{G}\left(j\right)}{2m}\;\text{otherwise}. \end{array} \end{array}$$ (16)

$$\begin{array}{c} Q_{0}\left(G,\Gamma \right)=\sum_{\gamma \in \Gamma }\sum_{i\neq j\in \gamma }\text{Conf}\left(G,i,j\right) \end{array}$$ (17)

$$\begin{array}{c} Q_{\text{Conf}}^{\tau }\left(G,\Gamma \right)=\tau .Q_{1}\left(G,\Gamma \right)+\left(1-\tau \right).Q_{0}\left(G,\Gamma \right) \end{array}$$ (18)

In Eq 16, with regard to a Graph *G*, the term $\left\{ \begin{array}{c} +1\;\text{if}\;\{i,j\}\in E,\\ -1\;\text{otherwise}. \end{array}\right.$ is a **local** (**microscopic**) vertices closeness measure, and the term $\frac{d_{G}\left(i\right).d_{G}\left(j\right)}{2m}$ is a **global (macroscopic)** vertices closeness measure, when in Eq 17, the term *Conf*(*G*, *i*, *j*) is an **intermediate local/global (mesoscopic)** vertices closeness measure.

Therefore in Eq 18, $Q_{Conf}^{\tau }\left(G,\Gamma \right)$ gives a weight of *τ* to the microscopic and macroscopic structure of Γ with regards to the Graph *G* and a weight of (1 − *τ*) to the mesoscopic structure. The closer the *τ* ∈ [0, 1] parameter is to 1, the less *Confluence* is taken into account.

## 4 Optimality

A Partitional Clustering **Δ is optimal** for a quality function *Q* **iff** for all partitioning Γ of *V*, *Q*(*G*, Δ)) ≧ *Q*(*G*, Γ)). Computing a Δ that maximizes $Q_{P_{edge}}\left(G,\Delta \right)$ is NP-complete [48], and the same holds for computing a Clustering that maximizes $Q_{Conf}^{\tau }$. However, when the number of vertices of a Graph *G* = (*V*, *E*) is small, the problem of maximizing the modularity can be turned into a reasonably tractable Integer Linear Program (see [48]): We define *n*^2^ decision variables *X*_*ij*_ ∈ {0, 1}, one for each pair of vertices {*i*, *j*} ∈ *V*. The key idea is that we can build an equivalence relation on *V* (*i* ∼ *j* iff *X*_*ij*_ = 1) and therefore a partitioning of *V*. To guarantee that the decision variables give rise to an equivalence relation, they must satisfy the following constraints:

**Reflexivity**: ∀*i* ∈ *V*, *X*_*ii*_ = 1;
**Symmetry**: ∀*i*, *j* ∈ *V* : *X*_*ij*_ = *X*_*ji*_;
**Transitivity**: $\forall i,j,k\in V:\{\begin{array}{c} \forall i,j,k\in V:X_{ij}+X_{jk}-2.X_{ik}\leq 1;\\ \forall i,j,k\in V:X_{ik}+X_{ij}-2.X_{jk}\leq 1;\\ \forall i,j,k\in V:X_{jk}+X_{ik}-2.X_{ij}\leq 1. \end{array}$

With the following objective functions to maximize:

$$\begin{array}{c} \text{For}\;Q_{P_{\text{edge}}}:\sum_{i,j\in V}\;X_{ij}\{\begin{array}{c} 1-\frac{d_{G}\left(i\right).d_{G}\left(j\right)}{2m}\;\text{if}\;\left\{i,j\right\}\in E,\\ -\frac{d_{G}\left(i\right).d_{G}\left(j\right)}{2m}\;\text{otherwise}. \end{array} \end{array}$$ (19)

$$\begin{array}{c} \text{For}\;Q_{\text{Conf}}^{\tau }\;:\sum_{i\neq j\in V}\;X_{ij}.\{\begin{array}{c} \left(1-\tau \right).Conf\left(G,i,j\right)+\tau -\tau .\frac{d_{G}\left(i\right).d_{G}\left(j\right)}{2m}\;\text{if}\;\left\{i,j\right\}\in E,\\ \left(1-\tau \right).Conf\left(G,i,j\right)-\tau -\tau .\frac{d_{G}\left(i\right).d_{G}\left(j\right)}{2m}\;\text{otherwise}. \end{array} \end{array}$$ (20)

**Fig 1:**
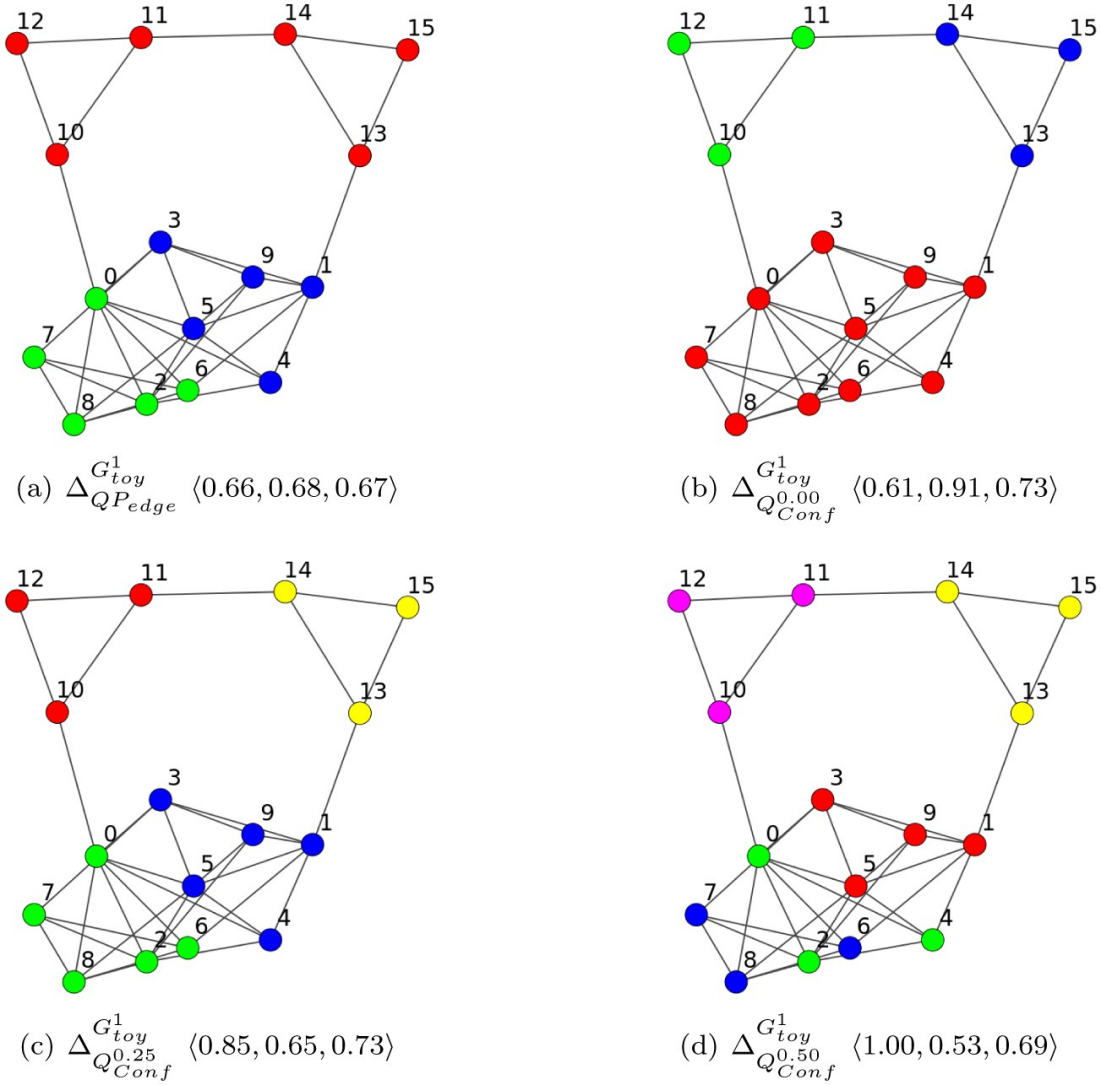
Optimal Clusterings for $Q_{P_{edge}}$ and *Q*_*Conf*_ on $G_{\text{toy}}^{1}$. If two vertices have same color, then they are in a same Module, with 〈**P, R, F**〉 where P=Precision(Pairs(Γ),E), R=Recall(Pairs(Γ),E), F=Fscore(Pairs(Γ),E).

The method *SGC* described in Algorithm 1 do not optimize a quality function, and the quality function used by *Infomap* can not be expressed as $\sum_{\gamma \in \Gamma }\;\sum_{i,j\in \gamma }\;sim\left(G,i,j\right)$, with *sim*(*G*, ., .) an R-valued symmetric similarity measure between vertices of *G*. We therefore left out this functions in our study of optimality, not having the ability to define their corresponding objective function to maximize in a similar fashion to what was done for $Q_{P_{edge}}$ and $Q_{Conf}^{\tau }$ with the formulas 19 and 20. In Fig 1, on a small artificial Graph $G_{toy}^{1}$, we compare the optimal Clusterings $\Delta_{Q_{P_{edge}}}^{G_{toy}^{1}}$, $\Delta_{Q_{Conf}^{0.00}}^{G_{toy}^{1}}$, $\Delta_{Q_{Conf}^{0.25}}^{G_{toy}^{1}}$ and $\Delta_{Q_{Conf}^{0.50}}^{G_{toy}^{1}}$ (with the Graph $G_{toy}^{1}$, if 0.50 < *x* < 1 then $\Delta_{Q_{Conf}^{\tau =x}}^{G_{toy}^{1}}=\Delta_{Q_{Conf}^{0.50}}^{G_{toy}^{1}}$) where:

-
$\Delta_{Q_{P_{\text{edge}}}}^{G_{\text{toy}}^{1}}=\{\delta_{Q_{P_{edge}}}^{1}=\left\{0,2,6,7,8\right\}$ , $\;\delta_{Q_{P_{edge}}}^{2}=\left\{1,3,4,5,9\right\}$, $\delta_{Q_{P_{edge}}}^{3}=\left\{10,11,12,13,14,15\right\}\}$;
-
$\Delta_{Q_{\text{Conf}}^{0.0}}^{G_{\text{toy}}^{1}}=\{\delta_{Q_{Conf}^{0.0}}^{1}=\left\{0,1,2,3,4,5,6,7,8,9\right\}$ , $\delta_{Q_{Conf}^{0.0}}^{2}=\left\{10,11,12\right\}$, $\delta_{Q_{Conf}^{0.0}}^{3}=\left\{13,14,15\right\}\}$;
-
$\Delta_{Q_{\text{Conf}}^{0.25}}^{G_{\text{toy}}^{1}}=\{\delta_{Q_{Conf}^{1.0}}^{1}=\left\{0,2,6,7,8\right\}$ , $\;\delta_{Q_{Conf}^{1.0}}^{2}=\left\{1,3,4,5,9\right\}$, $\delta_{Q_{Conf}^{1.0}}^{3}=\left\{10,11,12\right\}$, $\delta_{Q_{Conf}^{1.0}}^{4}=\left\{13,14,15\right\}\}$;
-
$\Delta_{Q_{\text{Conf}}^{0.50}}^{G_{\text{toy}}^{1}}=\{\delta_{Q_{Conf}^{0.50}}^{1}=\left\{0,2,4\right\}$ , $\delta_{Q_{Conf}^{0.50}}^{2}=\left\{1,3,5,9\right\}$, $\delta_{Q_{Conf}^{0.50}}^{3}=\left\{6,7,8\right\}$, $\;\delta_{Q_{Conf}^{0.50}}^{4}=\left\{10,11,12\right\}$, $\;\delta_{Q_{Conf}^{0.50}}^{5}=\left\{13,14,15\right\}\}$.

We can already notice that growing *τ* does not imply a simple splitting of the Modules (an approach that would be hierarchical), which we can see by going from *τ* = 0.25 to *τ* = 0.50 where there is no $\delta_{Q_{Conf}^{0.25}}$ such that $\delta_{Q_{Conf}^{0.50}}^{1}=\left\{0,2,4\right\}\;\subseteq \;\delta_{Q_{Conf}^{0.25}}$.

## 5 Binary edge-classifier by nodes blocks

What metric to use to estimate the accuracy of the four Clusterings in Fig 1? Much literature addresses this fundamental question [49–51]. Here we propose the definition of **B***inary* **E***dge-* **C***lassifier* **B***y nodes* **B***locks* (*BECBB*). To measure the quality of a Clustering Γ on a Graph *G* = (*V*, *E*), an intuitive, simple and efficient approach is to consider a Clustering Γ (with or witout overlaps), as a *BECBB* trying to predict the edges of a Graph: classifying each pairs of vertices into two classes, the *PositiveEdge* and the *NegativeEdge*.

**Definition**: A BECBB is a pairs of nodes binary classifier trying to predict the edges of a Graph. It is not allowed to give two complementary sets of pairs of nodes, one for its predictions as *PositiveEdge* and its complementary set for its predictions as *NegativeEdge*, but is forced to provide its predictions in the form of nodes blocks *B*_*i*_ ⊆ *V*: classifying as *PositiveEdge* a pair {*x*, *y*} if ∃*i* such *x*, *y* ∈ *B*_*i*_ else classifying it as *NegativeEdge*. If blocks are allowed to overlap then it is a *BECBB*^*OV*^ else it is a *BECBB*^*NO*^.

Let Γ a Clustering (with or witout overlaps) of a Graph *G* = (*V*, *E*)

$$\begin{array}{c} Pairs\left(\Gamma \right)=\underset{\gamma \in \Gamma }{⋃}\;P_{2}^{\gamma }; \end{array}$$ (21)

Pairs(Γ)⋂E=TP(Γ,E) are the True Postives of Γ according to *E*;

$\bar{Pairs(\Gamma )\;⋃\;E}=\text{TN}(\Gamma ,E)$ are the True Negatives;

$Pairs\left(\Gamma \right)\;⋂\;\bar{E}=\text{FP}(\Gamma ,E)$ are the False Postives;

$\bar{Pairs(\Gamma )}\;⋂\;E=\text{FN}(\Gamma ,E)$ are the False Negatives.

We can then measure the Γ’s accuracy with the classical measures in diagnostic binary Classification [52, 53]:

$$\begin{array}{c} Precision\left(Pairs\left(\Gamma \right),E\right)=\frac{\left|TP(\Gamma ,E)\right|}{\left|Pairs(\Gamma )\right|}\in \left[0,1\right]; \end{array}$$ (22)

$$\begin{array}{c} Recall\left(Pairs\left(\Gamma \right),E\right)=\frac{\left|TP(\Gamma ,E)\right|}{\left|E\right|}\in \left[0,1\right]; \end{array}$$ (23)

$$\begin{array}{c} Fscore\left(Pairs\left(\Gamma \right),E\right)=2.\frac{Precision(Pairs(\Gamma ),E).Recall(Pairs(\Gamma ),E)}{Precision(Pairs(\Gamma ),E)+Recall(Pairs(\Gamma ),E)}\in \left[0,1\right]. \end{array}$$ (24)

We can use these three measures indifferently on Clusterings with or without overlaps, because the Eq 21 makes sense with Clusterings with or without overlaps.

**BECBB**^**OV**^: For any Graph *G* = (*V*, *E*), the set of all edges Γ = *E* can be considered as a *BECBB*^*OV*^. Then Γ = *E* is optimal because: *Prec*(Γ = *E*, *E*) = 1 (Γ does not include any non-edge in its Modules) and *Rec*(Γ = *E*, *E*) = 1 (Γ include all the edges in its Modules). It is also true for Γ = *The set of all the maximal cliques*.

**BECBB**^**NO**^: The metric Precision(Pairs(Γ=Method(G)),E) measures the ability of a *Method* not to include non-edges in the Modules it returns, whereas the metric Recall(Pairs(Γ=Method(G)),E) measures its ability to include the edges in the Modules it returns. For a *BECBB*^*NO*^, a good *Precision* and a good *Recall* are two ability that oppose each other (because a *BECBB*^*NO*^ is forced to provide its classifications in the form of blocks *B*_*i*_ without overlaps) but are simultaneously bothtogether desirable for a good Clustering method. The whole point of a good Clustering method, as *BECBB*^*NO*^, is therefore to favor *Precision* without disfavoring *Recall* too much or even favoring *Recall* without disfavoring the *Precision* too much, that is what the metric Fscore(Pairs(Γ=Method(G)),E) measures (it is the harmonic mean of *Precision* and *Recall*).

### 5.1 Properties

**Fig 2:**
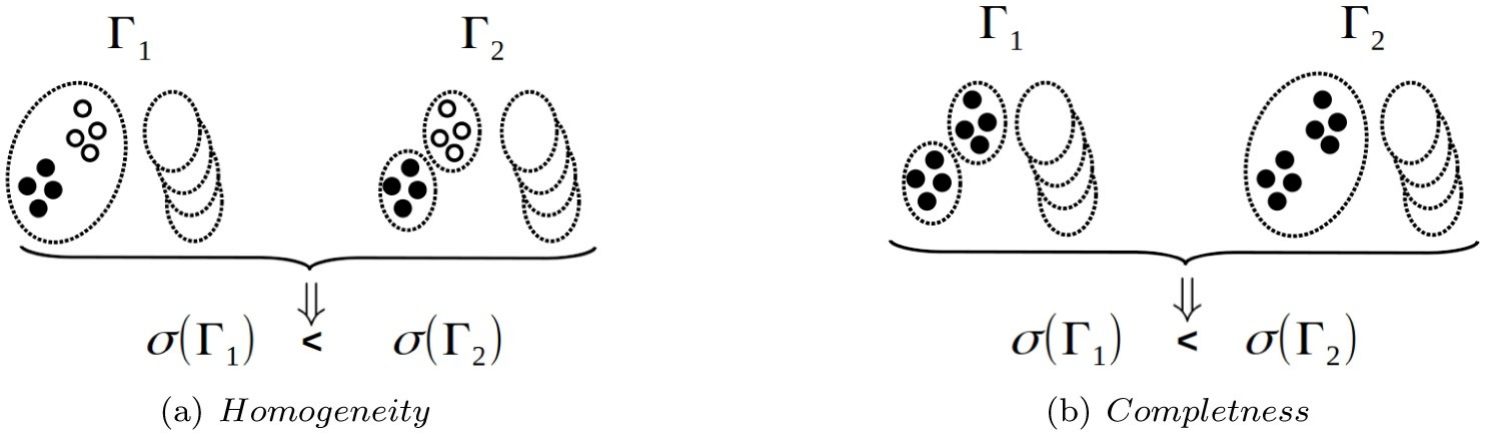
Homogeneity and Completeness: {*x*, *y*} ∈ *E* iff *x* and *y* have same color.

As showed in [51], it is better that a metric *σ*(Γ), to estimate the accuracy of a Clustering Γ, has the *Homogeneity* and *Completeness* [50] properties (see Fig 2 inspired by Figs 1 and 3 in [51]).

**Fig 3:**
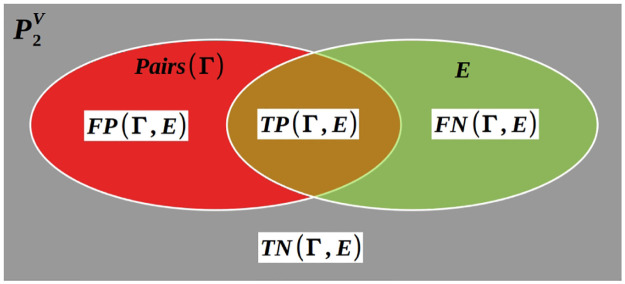
Binary classifiers of nodes pairs by nodes blocks.

- A metric *σ* to estimate the accuracy of Clusterings, has the *Homogeneity* property iff:
  $$\begin{array}{c} \begin{array}{c} TP\left(Pairs\left(\Gamma_{1}\right),E\right)=TP\left(Pairs\left(\Gamma_{2}\right),E\right)\\ \;\;\;\;\text{and}\\ FP\left(Pairs\left(\Gamma_{1}\right),E\right)>FP\left(Pairs\left(\Gamma_{2}\right),E\right) \end{array}\}\begin{array}{c} \;\\ \Rightarrow \;\sigma \left(\Gamma_{1}\right)<\sigma \left(\Gamma_{2}\right)\\ \; \end{array} \end{array}$$ (25)
- A metric *σ* to estimate the accuracy of Clusterings, has the *completeness* property iff:
  $$\begin{array}{c} \begin{array}{c} TP\left(Pairs\left(\Gamma_{1}\right),E\right)<TP\left(Pairs\left(\Gamma_{2}\right),E\right)\\ \;\;\;\;\text{and}\\ FP\left(Pairs\left(\Gamma_{1}\right),E\right)=FP\left(Pairs\left(\Gamma_{2}\right),E\right) \end{array}\}\begin{array}{c} \;\\ \Rightarrow \;\sigma \left(\Gamma_{1}\right)<\sigma \left(\Gamma_{2}\right)\\ \; \end{array} \end{array}$$ (26)

It is clear that the metric Fscore(Pairs(Γ),E) has these two properties, for any Clustering Γ with or without overlaps. Moreover the metric Fscore(Pairs(Γ),E) is independent of any extrinsic expectation to the Graph, we only need to trust the Graph itself. It is a good objective way to evaluate and compare Clusterings. So, to estimate the accuracy of Clustering methods *Method*_*i*_ and compare them on a Graph *G* = (*V*, *E*), we will use the three metrics:

**Precision(Method_i_(G = (V, E)), E)**: Measuring the ability of the *Method*_*i*_ not to include non-edges in the Modules it returns;
**Recall(Method_i_(G = (V, E)), E)**: Measuring its ability to include the edges in the Modules it returns;
**Fscore(Method_i_(G = (V, E)), E)**: Measuring the harmonic mean of its *Precision* and *Recall*.

Fig 1 shows the accuracy of $\Delta_{Q_{P_{edge}}}^{G_{toy}^{1}}$, $\Delta_{Q_{Conf}^{0.00}}^{G_{toy}^{1}}$, $\Delta_{Q_{Conf}^{0.25}}^{G_{toy}^{1}}$ and $\Delta_{Q_{Conf}^{0.50}}^{G_{toy}^{1}}$ considering these Clusterings as *BECBB*.

## 6 *Starling*, a heuristic for maximizing ${\text{Q}}_{\text{Conf}}^{\tau }$

In this section we describe *Starling*, a heuristic for tentatively maximizing $Q_{Conf}^{\tau }$. *Confluence* gives us an ordering on the edges of the Graph *G* = (*V*, *E*), in particular, sorting the edges {*i*, *j*} ∈ *E* by descending *Confluence*, forms the basis of a new Module merging strategy, described in Algorithm 2, intended to optimize $Q_{Conf}^{\tau }$.

**Algorithm 2** *Starling*: Graph Partitional Clustering

**Input**:

 *G* = (*V*, *E*) an undirected Graph

 *τ* ∈ [0, 1]

**Output**: *C*_*out*_ a Partitional Clustering of *G*

X↢{{i,j}∈Esuchi≠j}

**for** *i* ∈ *V* **do**   ▸ Initialization

 *mod*_*i*_ ↢ {*i*}   ▸ One vertex per Module

 *M*_*i*_ ↢ *i*   ▸ Vertex *i* is in Module *i*

ϒ ↢ ⌀

**While** *Υ* ≠ *X* **do**

$\left\{i,j\right\}↢\underset{\{x,y\}\in X-ϒ}{\text{arg}\;\text{max}}\;Conf\left(G,\;x,\;y\right)$ ▸ Line 1: Strategy based on *Confluence*

 ϒ ↢ ϒ ∪ {{*i*, *j*}}

 **if** *M*_*i*_ ≠ *M*_*j*_ **then**   ▸ *mod*_*i*_ and *mod*_*j*_ have not yet been merged together

$profit↢\sum_{u\in mod_{i}}\;\sum_{v\in mod_{j}}\{\begin{array}{c} \left(1-\tau \right).Conf\left(G,u,v\right)+\tau .\left(+1-\frac{d_{G}\left(u\right).d_{G}\left(v\right)}{2m}\right)\;\text{if}\;\left\{u,v\right\}\in E,\\ \left(1-\tau \right).Conf\left(G,u,v\right)+\tau .\left(-1-\frac{d_{G}\left(u\right).d_{G}\left(v\right)}{2m}\right)\;\text{otherwise}. \end{array}$

  **if** 0 ≤ *profit* **then**

   *mod*_*i*_ ↢ *mod*_*i*_ ∪ *mod*_*j*_   ▸ *mod*_*j*_ merge with *mod*_*i*_ in *mod*_*i*_

   *mod*_*j*_ ↢ ⌀   ▸ *mod*_*j*_ is dead

   **for** *k* ∈ *V* **do**   ▸ Updating the membership list

    **if** *M*_*k*_ = *j*   ▸ Vertex *k* was in *mod*_*j*_

     *M*_*k*_ ↢ *i*   ▸ Vertex *k* is now in *mod*_*i*_

*C*_*out*_ ↢ ⌀

**for** *i* ∈ *V* **do**

 **if** *mod*_*i*_ ≠ ⌀   ▸ *mod*_*i*_ is alive

  *C*_*out*_ ↢ *C*_*out*_ ∪ {*mod*_*i*_}

**return** *C*_*out*_

Different edges {*i*_1_, *j*_1_} ∈ *E* and {*i*_2_, *j*_2_} ∈ *E* might happen to have the exact same *Confluence* value (*Conf*(*G*, *i*_1_, *j*_1_) = *Conf*(*G*, *i*_2_, *j*_2_)), making the process (in Line 1) non-deterministic in general, because of its sensitivity on the order in which the edges with identical *Confluence* values are processed. A simple solution to this problem is to sort edges by first comparing their *Confluence* values and then using the lexicographic order on the words *i*_1_*j*_1_ and *i*_2_*j*_2_ when *Confluence* values are strictly identical.

We coded this Algorithm in *C*^++^ and in the following we used this program to analyze *Starling*’s results. With $G_{toy}^{1}$, $Starling(\tau ,G_{toy}^{1})$ find the optimal Clusterings for $Q_{Conf}^{\tau }$: $Starling\left(0.00,G_{toy}^{1}\right)=\Delta_{Q_{Conf}^{0.00}}^{G_{toy}^{1}}$, $Starling\left(0.25,G_{toy}^{1}\right)=\Delta_{Q_{Conf}^{0.25}}^{G_{toy}^{1}}$, $Starling\left(0.50,G_{toy}^{1}\right)=\Delta_{Q_{Conf}^{0.50}}^{G_{toy}^{1}}$.

## 7 Performance

In this section we estimate the accuracy of *Starling* and compare it with the methods *Louvain*, *Infomap* and *SGC*. We can Estimate the accuracy of Clustering Algorithms on:

**Real Graphs**: A set of *Terrain-Graphs* built from real data;
**A** ${\text{Benchmark}}_{B}$ : A set of computer-generated Graphs and its gold standard $\Gamma_{B}$ its expected Modules as expected overconnected regions.

Because we do not need to know *κ* the number of vertex groups in advance in the input of *Louvain* and *Infomap*, whereas we need it with *SGC*, for greater clarity, we compare on the one hand *Starling* versus *Louvain*, and *Infomap*, and on the other hand *Starling* versus *SGC*.

### 7.1 Starling versus Louvain and Infomap

#### 7.1.1 Performance on Real Terrain-Graphs

In this section we estimate the accuracy of Algorithms with three *Terrain-Graphs*:

- **G**_**Email**_: The Graph was generated using email data from a large European research institution [54, 55]. The Graph contains an undirected edge {*i*, *j*} if person *i* sent person *j* at least one email https://snap.stanford.edu/data/email-Eu-core.html.
- **G**_**DBLP**_: The DBLP computer science bibliography provides a comprehensive list of research papers in computer science [56]. Two authors are connected if they have published at least one paper together https://snap.stanford.edu/data/com-DBLP.html.
- **G**_**Amazon**_: A Graph was collected by crawling the Amazon website. It is based on the *Customers Who Bought This Item Also Bought* feature of the Amazon website [56]. If a product *i* is frequently co-purchased with product *j*, the Graph contains an undirected edge {*i*, *j*} https://snap.stanford.edu/data/com-Amazon.html.

Table 1 illustrates the pedigrees of these *Terrain-Graphs* and Table 2 shows the accuracies of *Louvain*, *Infomap* and *Starling* Considering each Clustering as a *BECBB*. We show also the number of Modules, the Length of the biggest Module and the computation time in seconds (All times are based on computations with a Quad Core Intel i5 and 32 Go RAM).

- **Louvain**: This is the fastest method, however its *Precision* is small, producing very few Modules, one of which is very large;
- **Infomap**: It gets a good *Fscore*, higher than this of *Louvain*.
- **Starling**^*τ*^: ∃*τ* ∈ [0, 1] such that *Starling*(*G*, *τ*) gets the highest *Fscore*. By default *τ* = 0.25 is a good compromise to obtain at the same time a good *Precision* and a good *Recall*. If we want to promote *Recall* (more edges in Modules) then we can decrease *τ*, and if we want to promote *Precision* (less non-edges in Modules) then we can increase *τ*.

**Table 1:** Pedigrees: *n* and *m* are the number of vertices and edges, 〈*k*〉 is the mean degree of vertices, *C* is the Clustering coefficient of the Graph, *L*_*lcc*_ is the average shortest path length between any two nodes of the largest connected component (largest subGraph in which there exist at least one path between any two nodes) and *n*_*lcc*_ the number of vertices of this component, λ is the coefficient of the best fitting power law of the degree distribution and *r*^2^ is the correlation coefficient of the fit, measuring how well the data is modelled by the power law.

Graph	n	m	〈k〉	C	L_lcc_(n_lcc_)	λ(r^2^)
**G_Email_**	1005	16064	31.97	0.27	2.59(986)	−1.02(0.81)
**G_DBLP_**	317080	1049866	6.62	0.31	6.79(317080)	−2.71(0.95)
**G_Amazon_**	334863	925872	5.53	0.21	11.95(334863)	−2.81(0.93)

**Table 2:** Graph Clustering as BECBBs: With 〈P,R,F〉 where : P=Precision(Pairs(Γ),E), R=Recall(Pairs(Γ),E), F=Fscore(Pairs(Γ),E), with [N, M] where N is the Number of Modules of Γ, M the Length of the biggest Module of Γ, and with (T) the computation time in seconds of Γ.

Graph	G = G_Email_	G = G_DBLP_	G = G_Amazon_
**Louvain**	〈0.11, 0.62, 0.18〉	〈0.00, 0.84, 0.00〉	〈0.00, 0.94, 0.00〉
	[26, 334] (0*s*)	[212, 22422] (12*s*)	[237, 12810] (6*s*)
**Infomap**	〈0.13, 0.60, 0.21〉	〈0.13, 0.72, 0.22〉	〈0.11, 0.82, 0.20〉
	[43, 319] (0*s*)	[16997, 811] (2165*s*)	[17265, 380] (1567*s*)
**Starling^0.000^**	〈0.16, 0.57, 0.24〉	〈0.08, 0.70, 0.15〉	〈0.10, 0.80, 0.18〉
	[63, 213] (9*s*)	[20044, 433] (752*s*)	[20479, 486] (160*s*)
**Starling^0.125^**	〈0.18, 0.51, 0.27〉	〈0.10, 0.70, 0.17〉	〈0.11, 0.80, 0.20〉
	[72, 140] (5*s*)	[21809, 396] (714*s*)	[22400, 435] (147*s*)
**Starling^0.250^**	〈0.26, 0.45, 0.33〉	〈0.12, 0.69, 0.20〉	〈0.13, 0.78, 0.23〉
	[102, 98] (2*s*)	[24852, 296] (584*s*)	[25906, 374] (134*s*)
**Starling^0.375^**	〈0.36, 0.40, 0.37〉	〈0.16, 0.67, 0.26〉	〈0.17, 0.76, 0.28〉
	[154, 84] (1*s*)	[29545, 252] (465*s*)	[31374, 308] (121*s*)
**Starling^0.500^**	〈0.49, 0.35, 0.41〉	〈0.25, 0.63, 0.36〉	〈0.26, 0.72, 0.38〉
	[235, 72] (1*s*)	[40905, 171] (347*s*)	[44597, 199] (108*s*)
**Starling^0.625^**	〈0.63, 0.29, 0.40〉	〈0.61, 0.52, 0.56〉	〈0.52, 0.59, 0.55〉
	[284, 52] (1*s*)	[87286, 116] (298*s*)	[80104, 32] (101*s*)
**Starling^0.750^**	〈0.69, 0.27, 0.38〉	〈0.83, 0.45, 0.58〉	〈0.70, 0.49, 0.57〉
	[319, 47] (1*s*)	[121392, 116] (252*s*)	[115637, 19] (78*s*)
**Starling^0.875^**	〈0.75, 0.23, 0.35〉	〈0.87, 0.43, 0.58〉	〈0.75, 0.45, 0.56〉
	[327, 30] (0*s*)	[124338, 113] (246*s*)	[121999, 16] (74*s*)
**Starling^1.000^**	〈0.77, 0.22, 0.35〉	〈0.94, 0.40, 0.56〉	〈0.86, 0.38, 0.52〉
	[378, 30] (0*s*)	[142371, 113] (239*s*)	[153712, 13] (68*s*)

#### 7.1.2 Performance on Benchmark_ER_

*Benchmark*_*ER*_ is the class of Random Graphs studied by Erdös and Rényi [57, 58] with parameters *N* the number of vertices and *p* the connection probability between two vertices. Random Graphs do not have a meaningful group structure, and they can be used to test if the Algorithms are able to recognize the absence of Modules. Therefore, we set *N* = 128, and we will study the accuracy of the methods with *Benchmark*_*ER*_ according to *p*.

**Fig 4:**
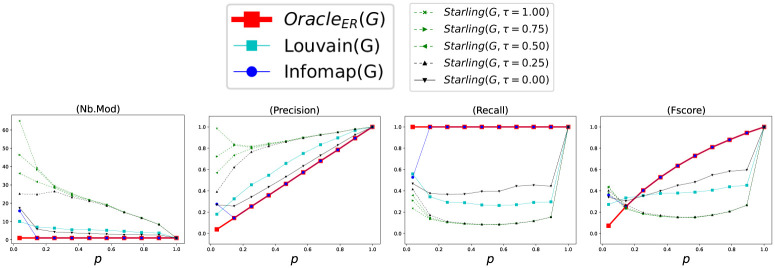
Performance with Benchmark_ER_. Each point (*x*, *y*) is the average over 100 Graphs with *p* = *x*.

Let $G_{ER}=\left(V_{G_{ER}},E_{G_{ER}}\right)$ a Random Graph built by *Benchmark*_*ER*_, Γ_*ER*_ = {*V*} with only one Module, and *Oracle*_*ER*_(*G*_*ER*_) = Γ_*ER*_ = {*V*} the Oracle’s method who knows Γ_*ER*_. Fig 4 shows the accuracy of the methods according to *p* considering each Clustering as a *BECBB*. We can see that:

- *Oracle*_*ER*_ knows Γ_*ER*_, but does not know the concretely constructed edges $E_{G_{ER}}$. Its number of Modules is always = 1. Its *Precision* increases when *p* increases, because *density* increases. Its *Recall* is always = 1. Its *Fscore* increase;
- The best *Precision*s are done with *Starling*^*τ*=0.25^ (but with a lot of Modules);
- The best *Recall*s are done with *Infomap*;
- The best *Fscore*s are done with *Infomap*, except while *p* <≈ 0.2, then it is with *Starling*^*τ*=0.25^;

*7.1.2.1 Starling detects the slightly-overconnected regions*. To observe more closely the behavior of *Starling*, we draw at Random one of the 100 Graphs with *p* = 0.25 which made it possible to construct the Fig 4. This Graph has a number of vertices *n* = 128, a number of edges *m* = 2077, a mean degree of vertices 〈*k*〉 = 32.45, a density *d* = 0.26, a Clustering coefficient *C* = 0.27, and a average shortest path length between any two nodes *L* = 1.74.

In this Random Graph with (*n* = 128, *d* = 0.26), *Starling*^*τ*=0.00^ finds four Modules *δ*_1_ with (*n*_1_ = 51, *d*_1_ = 0.33), *δ*_2_ with (*n*_2_ = 39, *d*_2_ = 0.35), *δ*_3_ with (*n*_3_ = 31, *d*_3_ = 0.34), *δ*_4_ with (*n*_4_ = 7, *d*_4_ = 0.76), where *n*_*i*_ are their number of vertices and *d*_*i*_ are their edge density. So, the four Modules *δ*_*i*_ found by *Starling*^*τ*=0.00^ have a density greater than the one of the entire Graph, specially for *δ*_4_: *d*_4_ = 0.76 > *d* = 0.26.

The phenomenon of overconnected regions is particulary clear in *Terrain-Graphs*, but also occur in Erdős-Rényi Random Graphs. Indeed such Graphs are not completely uniform, they present an *embryo* of structure with slightly-overconnected regions resulting from Random fluctuations (for exemple the Module *δ*_4_ which is clearly overconnected in this Graph).

It is these slightly-overconnected regions present in Random Graphs that are exploited and amplified in [59] to transform a Random Graph into a shaped-like *Terrain*-*Graph* and that *Starling* detects in a Random Graph, and so accepts as Modules (especially if *τ* increases). This is why in the Fig 4 the *Precision* of *Starling* is greater than that of *Oracle*_*ER*_. It is because the densities of the Modules found by *Starling* are greater than the density of the single Module *V* of *Oracle*_*ER*_ (which increases with *p*). However the number of edges between the Modules found by *Starling* remains large, this is why the *Recall* of *Starling* stays small (especially if *τ* increases).

*7.1.2.2* *Behavior*.

**(i)** *Infomap* usually returns Γ = {*V*}. Which means: **Infomap identify the absence of strong structures**;
**(ii)** *Starling*^*τ*^ returns Modules which have a density greater than the one of the entire Graph, the slightly-overconnected regions (especially if *τ* increases). Which means: **Starling^*τ*^ identifies the presence of weak structures**.

#### 7.1.3 Performance on Benchmark_LFR_

In most *Terrain-Graphs*, the distribution of degrees is well approximated by a power law. Similarly, in most *Terrain-Graphs*, the distribution of community sizes is well approximated by a power law [40, 60]. Therefore, in order to produce artificial Graphs with a meaningful group structure similar to most *Terrain-Graphs*, Lancichinetti, Fortunato and Radicchi proposed *Benchmark*_*LFR*_ [61] (Code to generate *Benchmark*_*LFR*_ Graphs can be downloaded from Andrea Lancichinetti’s homepage https://sites.google.com/site/andrealancichinetti/home). The Graphs in *Benchmark*_*LFR*_ are parameterized with:

- *N* their number of vertices;
- *k* their average degree;
- *γ* the power law exponent of their degree distribution;
- *β* the power law exponent of their community sizes distribution;
- *μ* ∈ [0, 1] their mixing parameter: Each vertex shares a fraction 1 − *μ* of its links with the other vertices of its community and a fraction *μ* with the other vertices of the Graph.

With *Benchmark*_*LFR*_, when the mixing parameter *μ* is weak, the overconnected regions are well separated from each other, and when *μ* increases, the overconnected regions are less clear. Therefore, we set *N* = 1000, and *k* = 15 or *k* = 25, and (*γ* = 2, *β* = 1) or (*γ* = 2, *β* = 2) or (*γ* = 3, *β* = 1) and for each of these six configurations, we will study the accuracy of the methods according to *μ*.

Let $G_{LFR}=\left(V_{G_{LFR}},E_{G_{LFR}}\right)$ a Graph built by *Benchmark*_*LFR*_, $\Gamma_{G_{LFR}}$ its expected Modules as expected overconnected regions, and $Oracle_{LFR}\left(G_{LFR}\right)=\Gamma_{G_{LFR}}$ the Oracle’s method which knows the $\Gamma_{G_{LFR}}$ of each *G*_*LFR*_.

**Fig 5:**
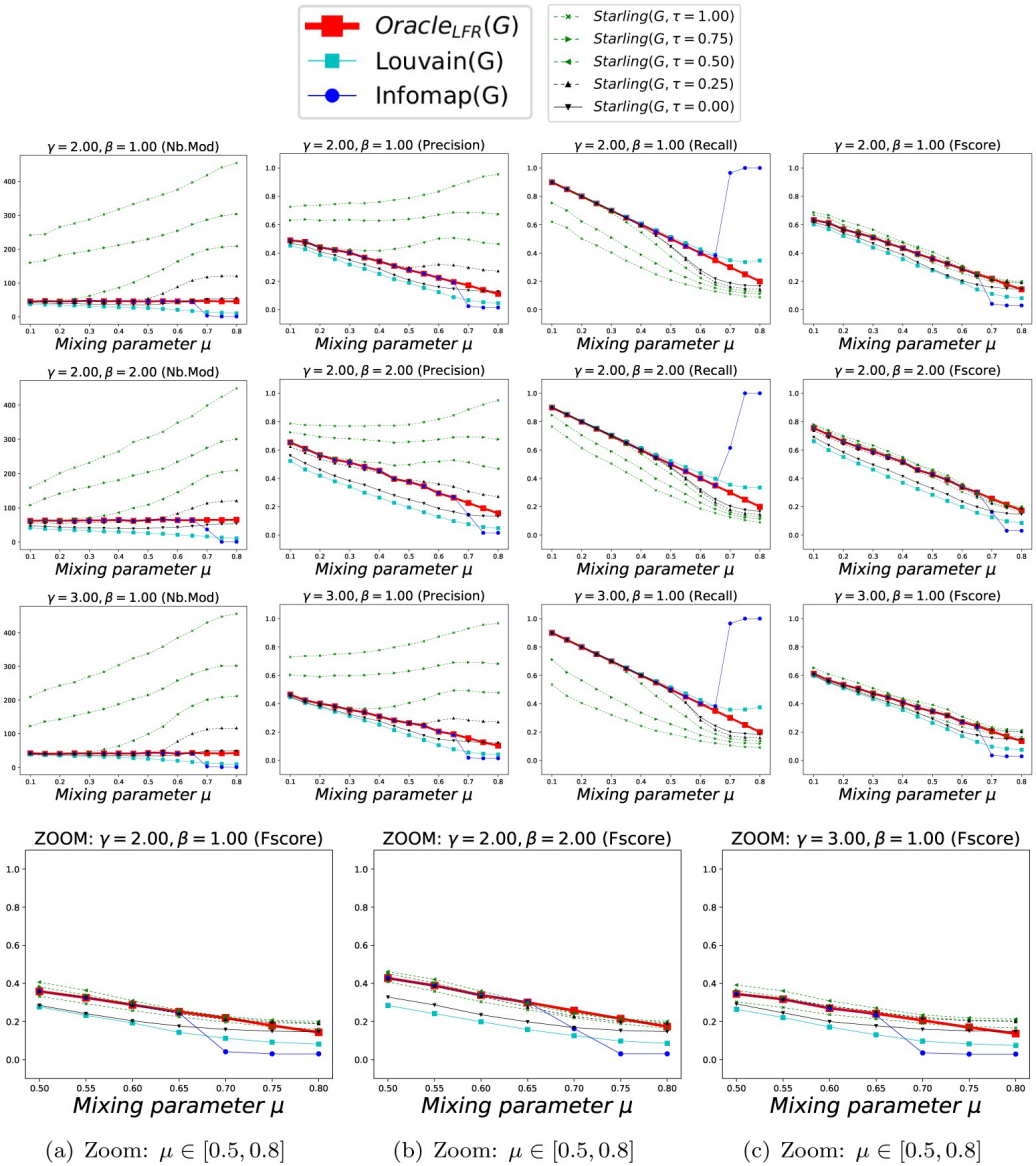
Performance with Benchmark_LFR_ (k = 15). Each point (*x*, *y*) is the average over 100 Graphs with *μ* = *x*. Fig 5(a)–5(c) are zooms on the *Fscore*s when the overconnected regions are less clear (i.e. when we can no longer trust $Oracle_{LFR}\left(G_{LFR}\right)=\Gamma_{G_{LFR}}$).

**Fig 6:**
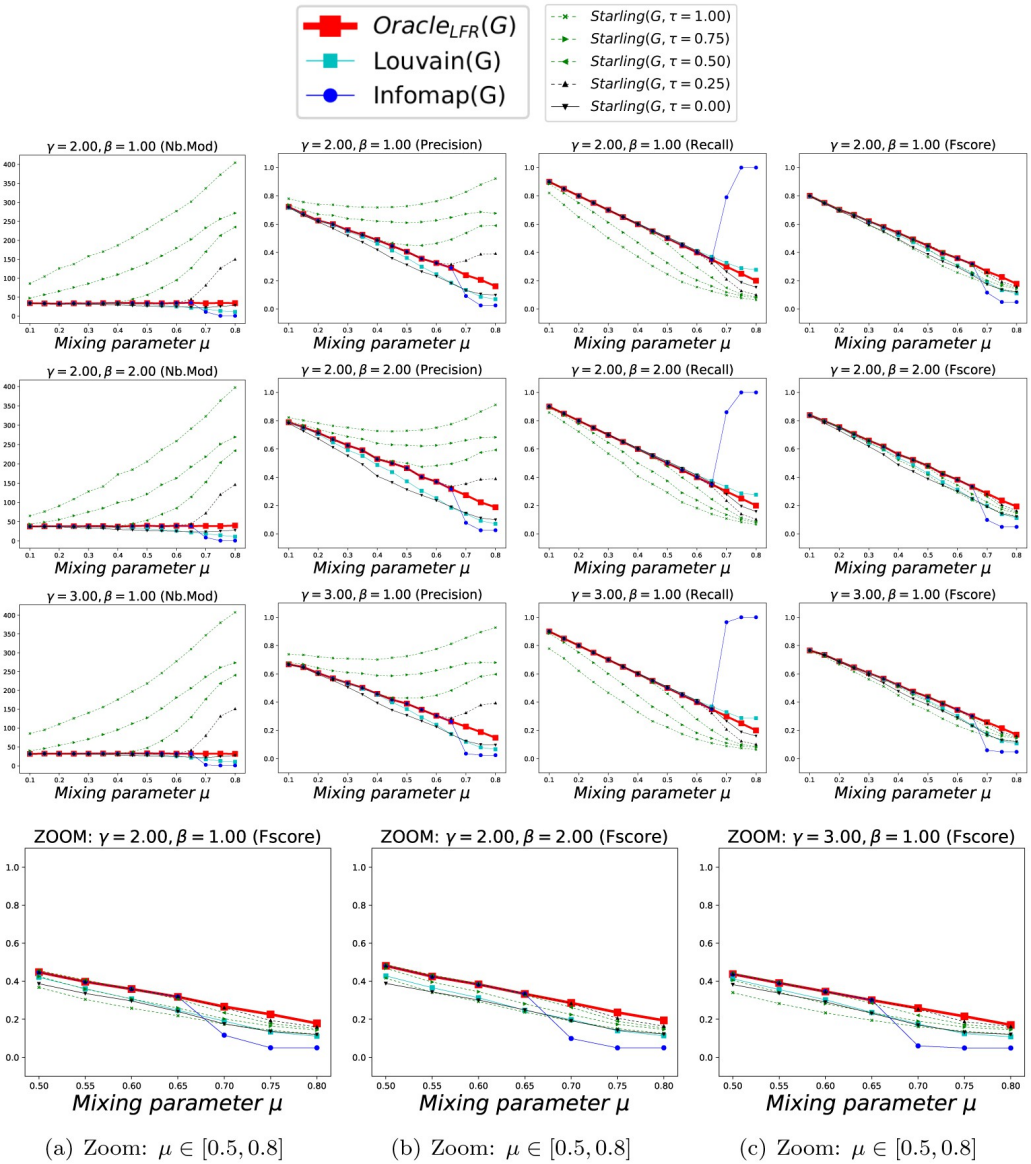
Performance with Benchmark_LFR_ (k = 25). Each point (*x*, *y*) is the average over 100 Graphs with *μ* = *x*. Fig 6(a)–6(c) are zooms on the *Fscore*s when the overconnected regions are less clear (i.e. when we can no longer trust $Oracle_{LFR}\left(G_{LFR}\right)=\Gamma_{G_{LFR}}$).

We show in Figs 5 and 6 the accuracy of the methods according to *μ*, considering each Clustering as a *BECBB*. We can see that:

- *Oracle*_*LFR*_ knows the $\Gamma_{G_{LFR}}$ of each *G*_*LFR*_, but does not know their concretely constructed edges $E_{G_{LFR}}$. Its number of Modules is always $|\Gamma_{G_{LFR}}|$. Its *Precision* decreases when *μ* increase, because there are more and more non-edges in the expected Modules, but *Oracle*_*LFR*_ does not know it. Its *Recall* decreases when *μ* increase, because there are more and more edges outside the expected Modules, but *Oracle*_*LFR*_ does not know it. Its *Fscore* decreases when *μ* increase, because its *Precision* and its *Recall* decreases;
- The best *Precision*s are done with *Starling*^*τ*=0.25^, but with a lot of Modules when the overconnected regions are less clear (because here again (see section 7.1.2.2) *Starling* identifies the presence of the large number of small slightly-overconnected regions as Modules present in these Graphs);
- The best *Recall*s are done with *Infomap*, but with very few Modules, and often only one, when the overconnected regions are less clear (because there is no way to compress the description of the path of a Random walker in these Graphs);
- The best *Fscore*s are done with *Infomap* and *Starling*^*τ*=0.25^ except when the overconnected regions are less clear, then it is with *Starling*^*τ*=0.25^.

### 7.2 Starling versus SGC

#### 7.2.1 Performance on Real Terrain-Graphs

In this section, we compare *Starling*(*G*, *τ*) with respect to *SGC*(*G*, *κ*), *κ* varying, on three little *Terrain-Graphs*:

- *G*_*Email*_: The Graph seen in section 7.1.1;
-
$G_{dblp_{811}}$ : The subGraph of *G*_*dblp*_ on the vertices of the larger Module of *Infomap*(*G*_*dblp*_) which has 811 vertices;
-
$G_{amazon_{380}}$ : The subGraph of *G*_*amazon*_ on the vertices of the larger Module of *Infomap*(*G*_*amazon*_) which has 380 vertices;

Table 3 illustrates the pedigrees of these *Terrain-Graphs*.

**Table 3:** Pedigrees: The notations are identical to those of Table 1.

Graph	n	m	〈k〉	C	L_lcc_(n_lcc_)	λ(r^2^)
**G_Email_**	1005	16064	31.97	0.27	2.59(986)	−1.02(0.81)
$G_{{\text{dblp}}_{811}}$ :	811	3774	9.31	0.19	3.33(811)	−1.35(0.91)
$G_{{\text{amazon}}_{380}}$	380	959	5.06	0.06	2.92(380)	−1.11(0.66)

The dataset describing *G*_*Email*_ contains “ground-truth” community memberships of the nodes $C:V_{G_{Email}}\to D$. Each individual belongs to exactly one of 42 departments *D* = {*d*_1_, …*d*_42_} at the research institute from which the emails are extracted. Let Γ_*Dep*_ the Gold-Standart partition of $V_{G_{Email}}$ such:

$$\begin{array}{c} \Gamma_{Dep}=\underset{d_{i}\in D}{⋃}\{\{j,\;\text{such}\;C\left(j\right)=d_{i}\}\} \end{array}$$

We can therefore evaluate the quality of a Clustering by partition on *G*_*Email*_ according to two kinds of truths:

**Intrinsic-Truth**: The edges of *G*_*Email*_ as we did in the previous sections with *Precision*, *Recall*, *Fscore* respectively defined by the formulas 22, 23 and 24;
**Extrinsic-Truth**: $Pairs(\Gamma_{Dep})$ by replacing *E* by $Pairs(\Gamma_{Dep})$ in the three formulas 22, 23 and 24.

**Fig 7:**
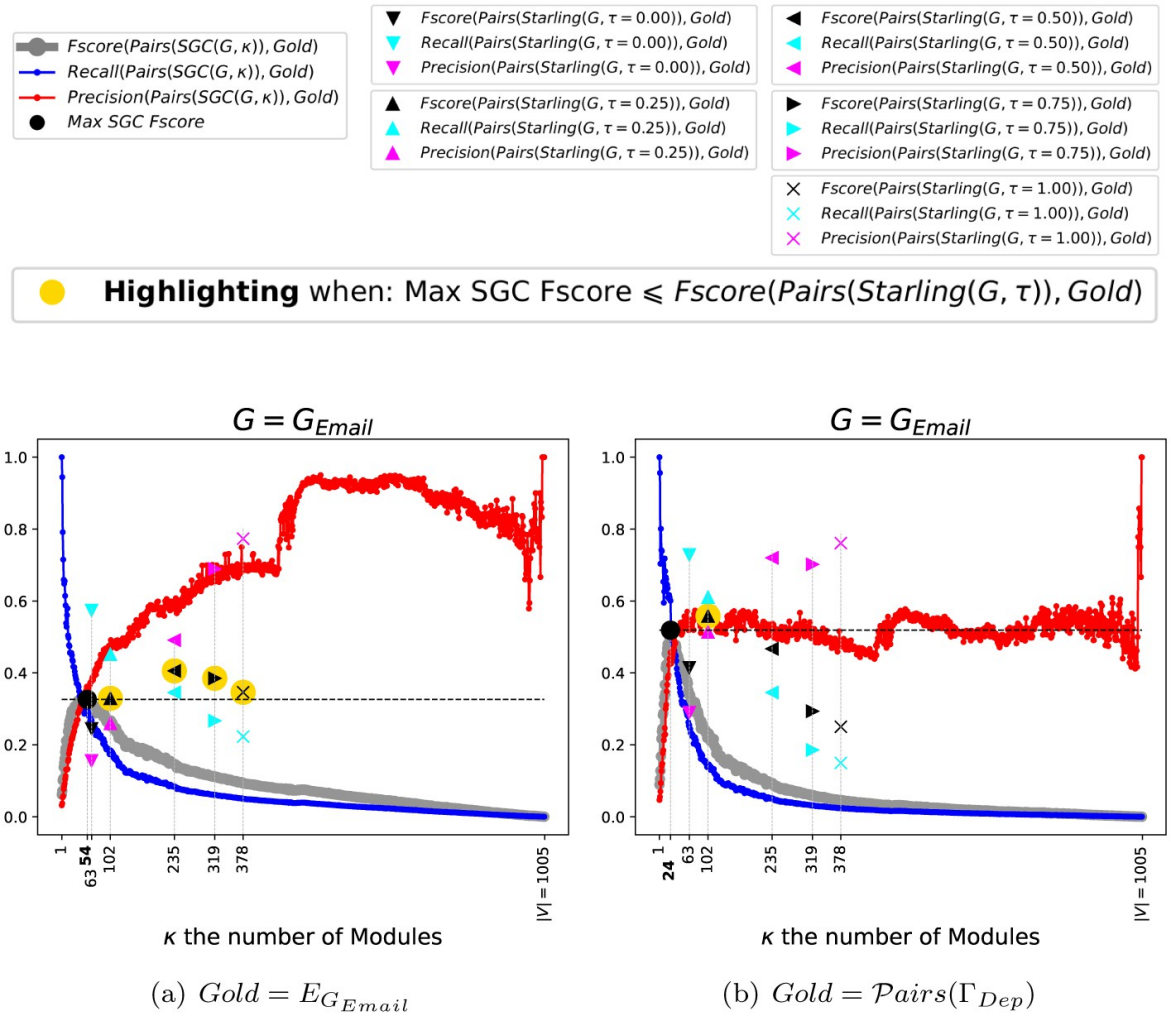
Performance of SGC(${\text{G}}_{\text{Email}}=({\text{V}}_{{\text{G}}_{\text{Email}}},{\text{E}}_{{\text{G}}_{\text{Email}}})$, *κ*), *κ* varying. According to the intrinsic truth $E_{G_{Email}}$ in Fig 7(a), and in Fig 7(b) according to the extrinsic truth $Pairs(\Gamma_{Dep})=\cup_{\gamma \in \Gamma_{Dep}}P_{2}^{\gamma }$.

Fig 7 shows the performances of *SGC*(*G*_*Email*_, *κ*), one hand according to the *Intrinsic*-*Truth* $E_{G_{Email}}$ in Fig 7(a), and on the other hand according to the *Extrinsic*-*Truth* $Pairs\left(\Gamma_{Dep}\right)={⋃}_{\gamma \in \Gamma_{Dep}}P_{2}^{\gamma }$ in Fig 7(b). We can see that:

- According to the *Intrinsic*-*Truth* $E_{G_{Email}}$ in Fig 7(a): *Starling*(*G*_*Email*_, *τ* = 0.25), with 102 Modules, gets *Precision* = 0.26, *Recall* = 0.45, *Fscore* = 0.33. The maximum *Fscore* of *SGC* is geted for *κ* = 54 with *Precision* = 0.36, *Recall* = 0.30, *Fscore* = 0.33. On the other hand for *τ* ∈ {0.50, 0.75, 1.00}, *Starling* gets a beter *Fscore* than the best *Fscore* of *SGC*.
  **As BECBB**: ∃*τ* ∈ [0, 1] such *Starling* gets a beter *Fscore* than the best *Fscore* of *SGC*.
- According to the *Extrinsic*-*Truth* $Pairs(\Gamma_{Dep})$ in Fig 7(b): *Starling*(*G*_*Email*_, *τ* = 0.25) gets *Precision* = 0.51, *Recall* = 0.61, *Fscore* = 0.56. The maximum *Fscore* of *SGC* is geted for *κ* = 24 with *Precision* = 0.46, *Recall* = 0.60, *Fscore* = 0.52.
  **According to** $P\text{airs}(\Gamma_{\text{Dep}})$ : ∃*τ* ∈ [0, 1] such *Starling* gets a beter *Fscore* than the best *Fscore* of *SGC*.

**Fig 8:**
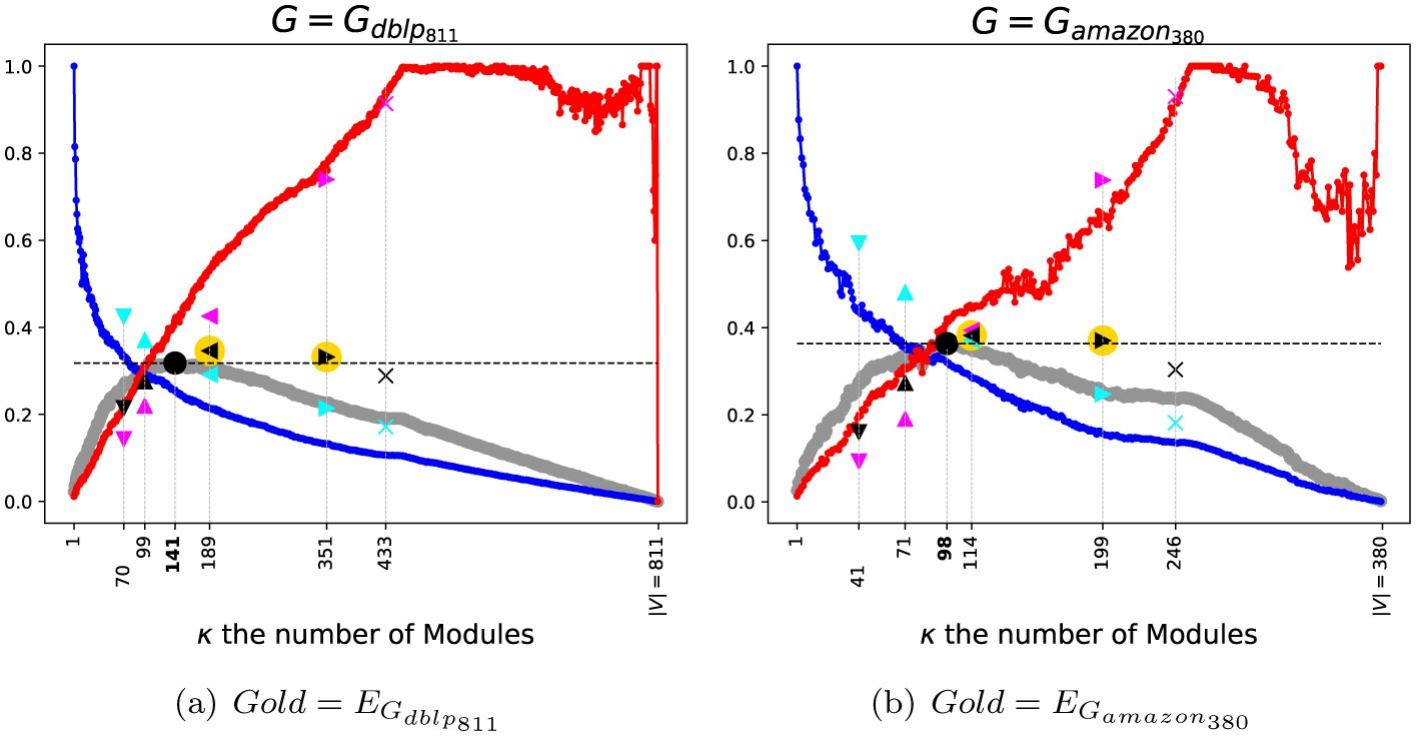
Performance of SGC(G = (V,E), *κ*) according to the intrinsic truth E, *κ* varying.

Fig 8 shows the performances as *BECBB*s of $SGC(G_{dblp_{811}},\kappa )$ and $SGC(G_{amazon_{380}},\kappa )$, according to their *Intrinsic*-*Truth* respectively $E_{G_{dblp_{811}}}$ and $E_{G_{amazon_{380}}}$. We can see that: ∃*τ* ∈ [0, 1] such *Starling* gets a beter *Fscore* than the best *Fscore* of *SGC*.

*7.2.1.1 Extrinsic-Truth according to Intrinsic-Truth of G_Email_*. Because $Precision(Pairs\left(\Gamma_{Dep}\right),E_{G_{Email}})=0.23$, $Recall(Pairs\left(\Gamma_{Dep}\right),E_{G_{Email}})=0.34$, $Fscore(Pairs\left(\Gamma_{Dep}\right),E_{G_{Email}})=0.27$, as *BECBB*, Γ_*Dep*_ is less efficient than *Starling*(*G*_*Email*_, *τ* = 0.25) or *SGC*(*G*_*Email*_, *κ* = 54) the best *BECBB* of *SGC*.

That is to say that Gold-Standards are not always the best *BECBB*s, we can not always trust Gold-Standards provided by Benchmarks or built using human assessors, which as showed in [62], generaly do not always agree with each other, even when their judgements are based on the same protocol.

In our present example with *G*_*Email*_, we can think that two individuals from the same department can communicate in real life more often than two individuals from different departments: *Two individuals from the same department do not necessarily need to communicate more by email than two individuals from different departments*.

#### 7.2.2 Performance on Benchmark_ER_

Because we need to know *κ* the number of groups of vertices in advance in the Input of *SGC*, to be able to compare *Starling* with *SGC* we define: *SGC*^*τ*^(*G*) = *SGC*(*G*, *κ* = |*Starling*(*G*, *τ*)|).

Let $G_{ER}=\left(V_{G_{ER}},E_{G_{ER}}\right)$ a Random Graph built by *Benchmark*_*ER*_, Γ_*ER*_ = {*V*} with only one Module, and *Oracle*_*ER*_(*G*_*ER*_) = Γ_*ER*_ = {*V*} the Oracle’s method who knows Γ_*ER*_.

**Fig 9:**
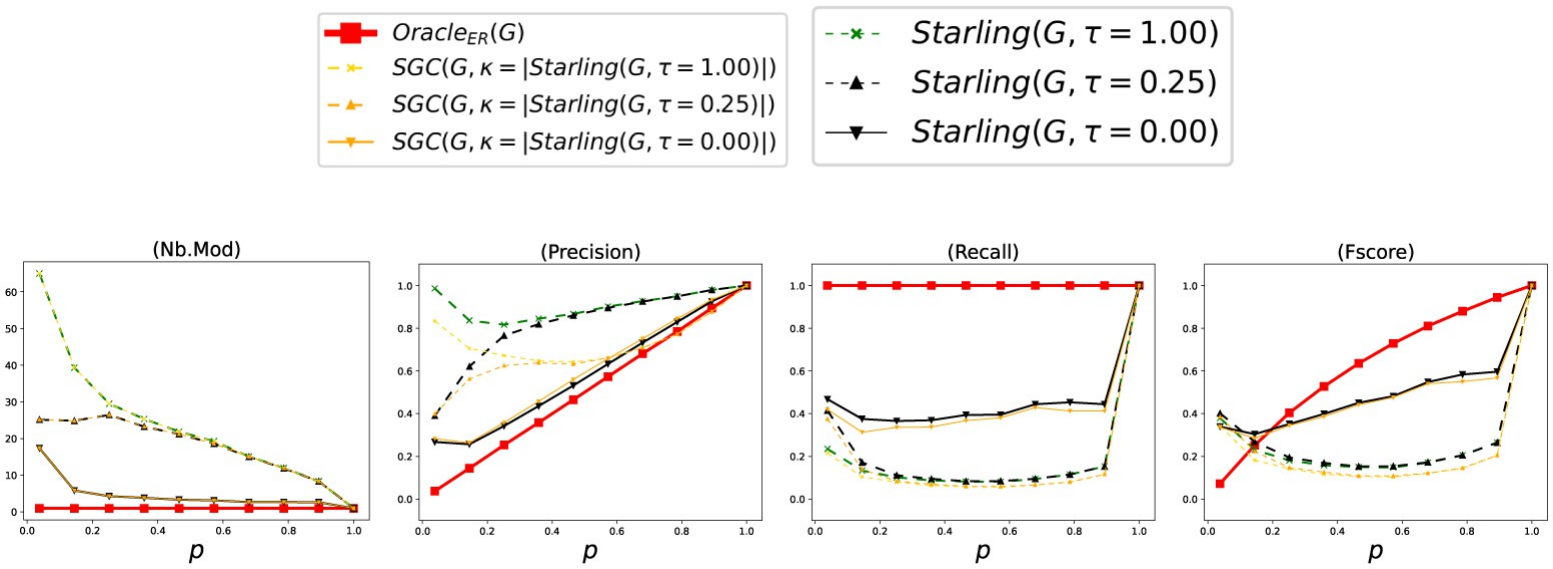
Performance with Benchmark_ER_. Each point (*x*, *y*) is the average over 100 Graphs with *p* = *x*.

Fig 9 shows the accuracy of the methods according to *p* considering each Clustering as a *BECBB*. We can see that: ∀*τ* ∈ {0.00, 0.25, 1.00} on these Figures, the *Fscore*s geted by *Starling*(*G*, *τ*) are always equal or greater than the *Fscore*s geted by *SGC*^*τ*^(*G*).

#### 7.2.3 Performance on Benchmark_LFR_

Let $G_{LFR}=\left(V_{G_{LFR}},E_{G_{LFR}}\right)$ a Graph built by *Benchmark*_*LFR*_, $\Gamma_{G_{LFR}}$ its expected Modules as expected overconnected regions, and $Oracle_{LFR}\left(G_{LFR}\right)=\Gamma_{G_{LFR}}$ the Oracle’s method which knows the $\Gamma_{G_{LFR}}$ of each *G*_*LFR*_.

**Fig 10:**
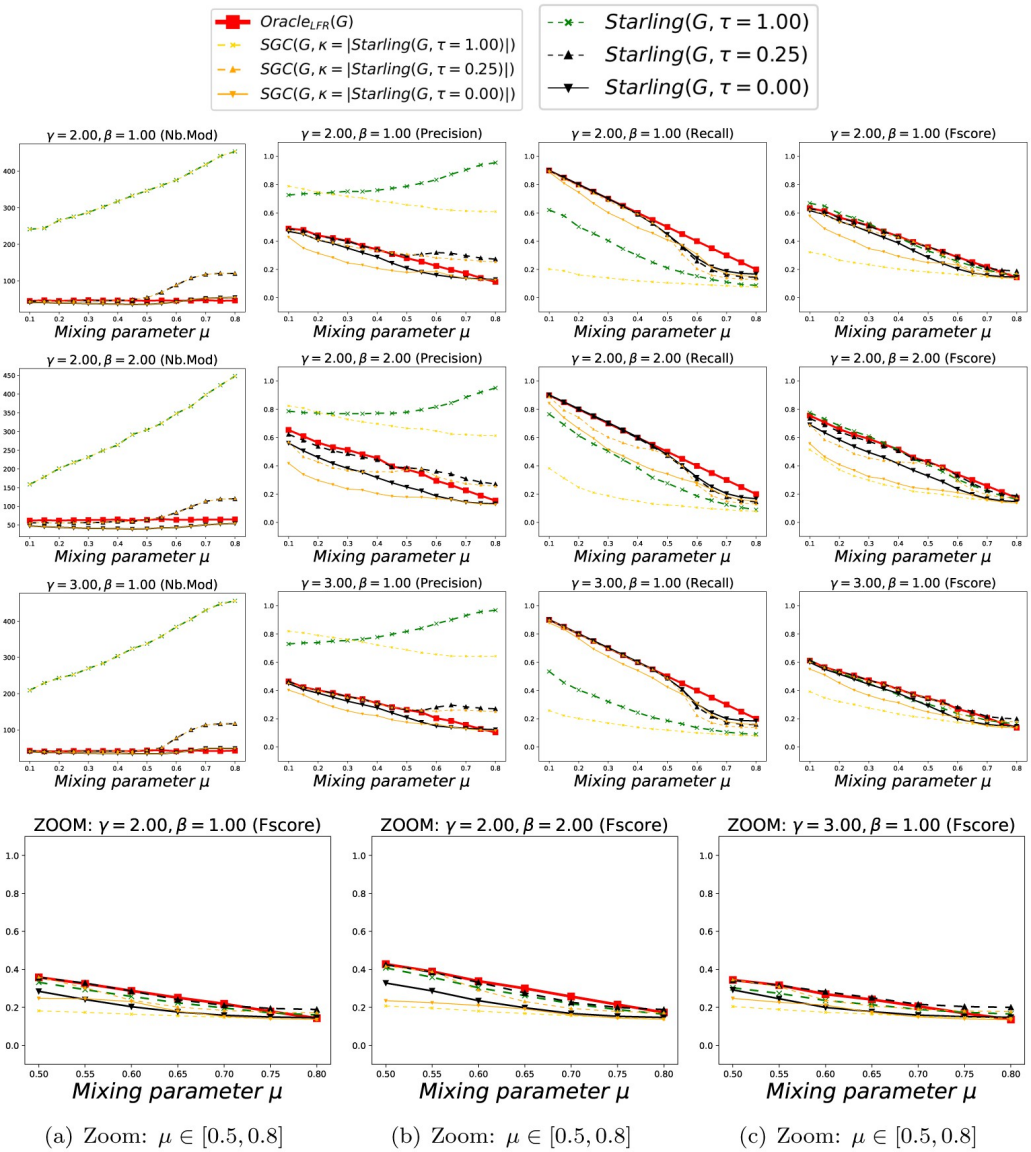
Performance with Benchmark_LFR_ (k = 15). Each point (*x*, *y*) is the average over 100 Graphs with *μ* = *x*. Fig 10(a)–10(c) are zooms on the *Fscore*s when the overconnected regions are less clear (i.e. when we can no longer trust $Oracle_{LFR}\left(G_{LFR}\right)=\Gamma_{G_{LFR}}$).

**Fig 11:**
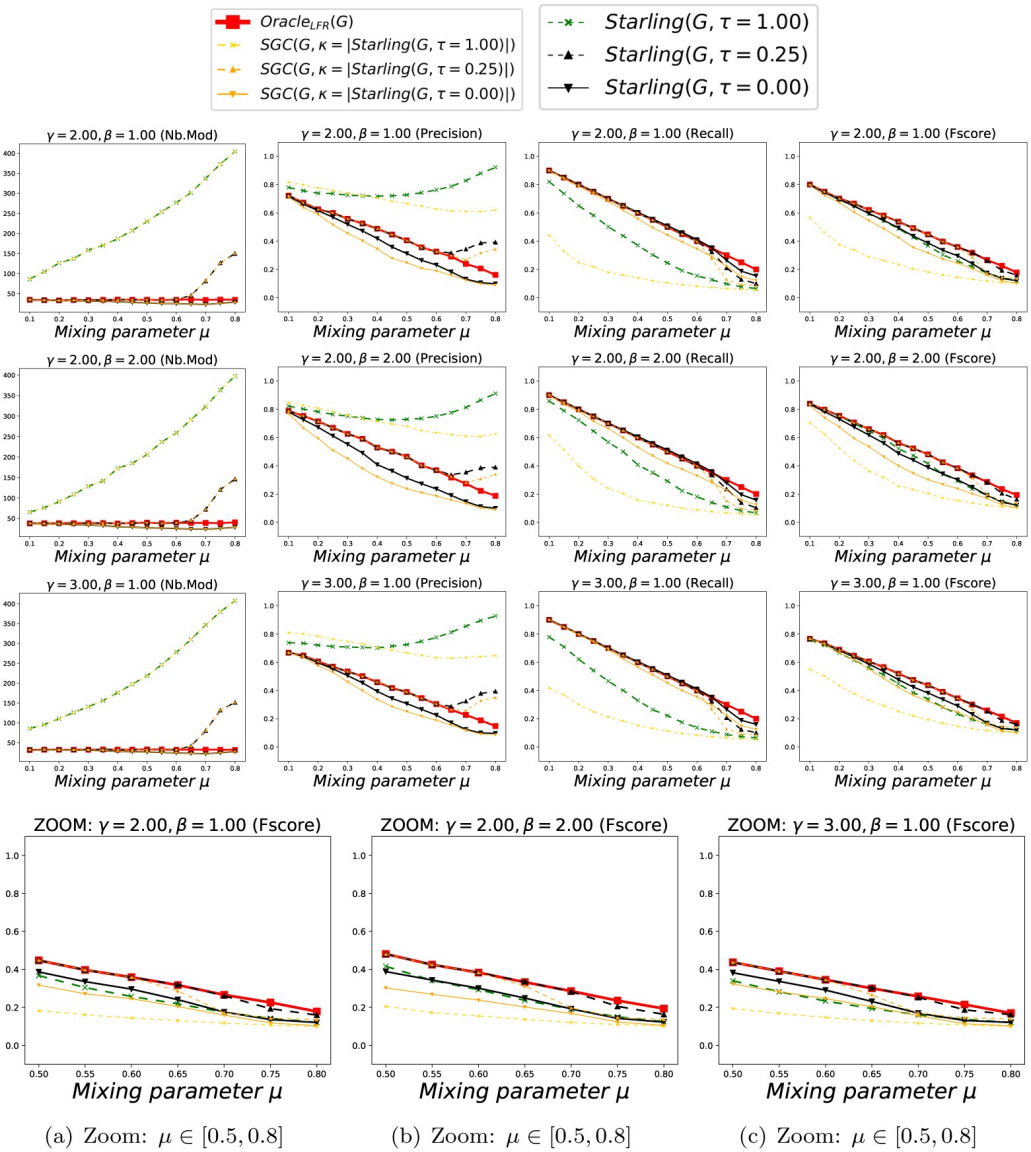
Performance with Benchmark_LFR_ (k = 25). Each point (*x*, *y*) is the average over 100 Graphs with *μ* = *x*. Fig 11(a)–11(c) are zooms on the *Fscore*s when the overconnected regions are less clear (i.e. when we can no longer trust $Oracle_{LFR}\left(G_{LFR}\right)=\Gamma_{G_{LFR}}$).

We show in Figs 10 and 11 the accuracy of methods *SGC*^*τ*^(*G*) and *Starling*(*G*, *τ*) according to *μ*, considering each Clustering as a *BECBB*. We can see that ∀*τ* ∈ {0.00, 0.25, 1.00} on these Figures, the *Fscore*s geted by *Starling*(*G*, 0.25) are always equal or greater than the *Fscore*s geted by *SGC*^*τ*^(*G*).

## 8 Discussion

### 8.1 Choosing the *τ* parameter of Starling

When using a Benchmark B to evaluate the performance of methods on a Graph $G_{B}=\left(V_{G_{B}},E_{G_{B}}\right)$, the Oracle’s method $Oracle_{B}$ knows the expected overconnected regions $\Gamma_{G_{B}}$ but do not knows the concretely constructed edges $E_{G_{B}}$. Therefore, when the overconnected regions are less clear, as *BECBB* (with $\text{Gold}=E_{G_{B}}$, Intrinsic-Truth), some methods may outperform the $Oracle_{B}$ method. This happens especially with the *Starling*^*τ*^ method if the *τ* parameter has been chosen appropriately.

We have seen in Formula 17 that the closer the *τ* ∈ [0, 1] parameter is to 1, the less *Confluence* is taken into account in $Q_{Conf}^{\tau }$. With *Terrain-Graphs*, we propose using *τ* = 0.25 as a first approach by default, then decreasing *τ* if we want to promote *Recall* (because it has the effect of decreasing the number of Modules and of increasing their sizes) or increasing *τ* if we want to promote *Precision* (because it has the effect of increasing the number of Modules and of decreasing their sizes).

### 8.2 Length of Random walks

For clarity and simplicity, we restricted the Random walks of $P_{G}^{t}\left(i⇝j\right)$ to a length of *t* = 3. A first study of the impact of the length of those Random walks to transform a Random Graph into a shaped-like *Terrain* − *Graph* was done in [59], but a deeper one should be carried to understand how the length influences the **meso**scopicity of *Confluence* and its effect on *Q*_*Conf*_ and *Starling*.

**Fig 12:**
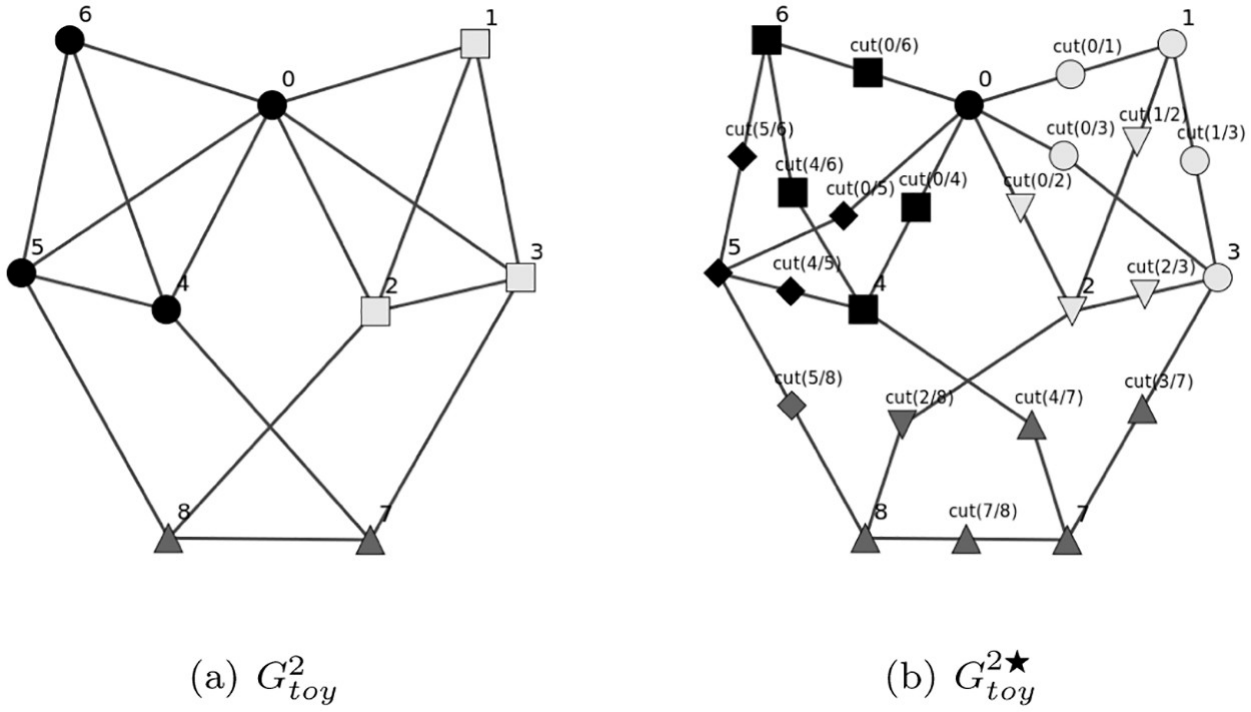
Optimal Clusterings for $Q_{Conf}^{0.0}$ with *t* = 3 and with *t* = 6: Shapes describe an optimal Clustering for $Q_{Conf}^{0.0}$ with *t* = 3, colors describe an optimal Clustering for $Q_{Conf}^{0.0}$ with *t* = 6.

For example we can build the Graph $G_{toy}^{2★}$ from $G_{toy}^{2}$ by inserting a new vertex in the middle of each edge. Fig 12 illustrates the optimal Clusterings on $G_{toy}^{2}$ and on $G_{toy}^{2★}$ for $Q_{Conf}^{0.0}$ with *t* = 3 and also with *t* = 6, allowing us to see that:

On ${\text{G}}_{\text{toy}}^{2★}$ with **t** = **6**:

$\delta_{Conf}^{★1}=\left\{0,4,5,6,cut\left(0/4\right),cut\left(0/5\right),cut\left(0/6\right),cut\left(4/5\right),cut\left(4/6\right),cut\left(5/6\right)\right\}$ ;

$\delta_{Conf}^{★2}=\left\{1,2,3,cut\left(0/1\right),cut\left(0/2\right),cut\left(0/3\right),cut\left(1/2\right),cut\left(1/3\right),cut\left(2/3\right)\right\}$ ;

$\delta_{Conf}^{★3}=\left\{7,8,cut\left(7/8\right),cut\left(3/7\right),cut\left(4/7\right),cut\left(2/8\right),cut\left(5/8\right)\right\}$ .

On ${\text{G}}_{\text{toy}}^{2}$ with **t** = **3**:

$\delta_{Conf}^{1}=\left\{0,4,5,6\right\}\subset \delta_{Conf}^{★1}$ ;

$\delta_{Conf}^{2}=\left\{1,2,3,\right\}\subset \;\delta_{Conf}^{★2}$ ;

$\delta_{Conf}^{3}=\left\{7,8\right\}\subset \delta_{Conf}^{★3}$ .

The length of Random walks *t* could be advantageously chosen taking into account *L*, the average number of edges on the shortest path between two vertices.

### 8.3 Directed graphs

If *G* is a positively weighted Graph by *W* = {*w*_*i*,*j*_ such {*i*, *j*} ∈ *E*}, then we can apply *Q*_*Conf*_ and *Starling* by replacing Eqs 7 and 10 by 27 and 28 respectively:

$$\begin{array}{c} \left[G\right]={\left(g_{i,j}\right)}_{i,j\in V}\;\;\text{with}\;\;g_{i,j}=\{\begin{array}{c} \frac{w_{i,j}}{\sum_{k\in V}w_{i,k}}\;\text{if}\;\left\{i,j\right\}\in E\text{,}\\ 0\;\text{otherwise}. \end{array} \end{array}$$ (27)

$$\begin{array}{c} Conf^{t}\left(G,i,j\right)=\{\begin{array}{c} 0\;\text{if}\;i=j,\\ \frac{P_{G}^{t}\left(i⇝j\right)-\frac{\sum_{k\in V}w_{k,j}}{\sum_{w\in W}w}}{P_{G}^{t}\left(i⇝j\right)+\frac{\sum_{k\in V}w_{k,j}}{\sum_{w\in W}w}}\;\text{otherwise}. \end{array} \end{array}$$ (28)

If *G* is a directed Graph, one can also consider using a variant of page rank [63–65] in place of Eq 8.

## 9 Conclusions and perspectives

In this paper, we defined *Confluence*, a mesoscopic vertex closeness measure based on short Random walks, which brings together vertices from the same overconnected region, and separates vertices coming from two distinct overconnected regions. Then we used *Confluence* to define $Q_{Conf}^{\tau }$, a new Clustering quality function, where the *τ* ∈ [0, 1] parameter is a handle on the *Precision* & *Recall*, the size and the number of Modules. With a small toy Graphs, we showed that optimal Clusterings for $Q_{Conf}^{\tau }$ improve the *Fscore* of the optimal Clusterings for *Modularity*.

We then introduced *Starling*(*G*, *τ*), a new heuristic based on the *Confluence* of edges designed to optimize $Q_{Conf}^{\tau }$ on a Graph *G*. On the same little toy Graph, we showed that *Starling*(*G*, *τ*) finds an optimal Clustering for $Q_{Conf}^{\tau }$.

Comparing *Starling*(*G*, *τ*) to *SGC*(*G*, *κ*), *Infomap*, and *Louvain* we show that:

- Performance with the *Terrain-Graphs* studied in this paper:
  *Louvain*(*G*): Returns Clusterings with a low *Fscore*, caused by a to much low *Precision* despite a large *Recall*;
  *Infomap*(*G*): Tends to favor *Recall* with good *Fscore*;
  *SGC*(*G*, *κ*): Returns Clusterings with a good *Fscore* if we know the good number of groups of vertices *κ* in advance;
  *Starling*(*G*, *τ*): Tends to favor *Precision* with good *Fscore*. ∃*τ* ∈ [0, 1] (usually around *τ* ≈ 0.25) such that the *Fscore* of the Clusterings returned by *Starling* is greater than the *Fscore*s of the Clusterings returned by *Infomap* and greater than the best *Fscore*s of the Clusterings returned by *SGC*.
- Performance with *Benchmark*_*ER*_:
  *SGC*(*G*, *κ* = |*Starling*(*G*, *τ*)|): *Fscore*(*SGC*(*G*, *κ* = |*Starling*(*G*, *τ*)|), {*V*}) ≈ *Fscore*(*Starling*(*G*, *τ*), {*V*});
  **(i)** *Infomap* usually returns Γ = {*V*}. Which means: *Infomap* identify the absence of strong structures;
  **(ii)** *Starling*^*τ*^ returns Modules which have a density greater than the one of the entire Graph, the slightly-overconnected regions (especially if *τ* increases). Which means: *Starling*^*τ*^ identifies the presence of weak structures.
- Performance with *Benchmark*_*LFR*_:
  - When the overconnected regions are clear: On the one hand, *Starling*(*G*, *τ* = 0.25) gets equivalent *Fscore*s than these of *Infomap* (see Figs 6 and 7). On the other hand *Starling*(*G*, *τ*) gets equivalent or greater *Fscore*s than these of *SGC*(*G*, *κ* = |*Starling*(*G*, *τ*)|) (see Figs 10 and 11);
  - When the overconnected regions become less clear, *Starling* favors *Precision* while *Infomap* favor *Recall*:
    **(1)** On the one hand, *Starling*(*G*, *τ* = 0.25) gets then greater *Fscore*s than these of *Infomap* (see Figs 5(a)–5(c) and 6(a)–6(c)). That’s because even in Non Erdös and Rényi Graphs, *Starling*^*τ*^ identifies the presence of weak structures thanks to its (*ii*) behavior, whereas *Infomap* identify the absence of strong structures because its (*i*) behavior.
      On the other hand, *Starling*(*G*, *τ* = 0.25) gets then equivalent or greater *Fscore*s than these of *SGC*(*G*, *κ* = |*Starling*(*G*, *τ* = 0.25)|) (see Figs 10(a)–10(c) and 11(a)–11(c)).
    **(2)** Often (*τ* dependent) *Starling*(*G*, *τ*), thanks to its (*ii*) behavior, is able to get larger *Fscore*s than these of *Oracles* that would only knows their expected overconnected regions (concretely slightly-overconnected), ignoring *E* their concretely constructed edges. *SGC*(*G*, *κ* = |*Starling*(*G*, *τ* = 0.25)|) can also succeed (see Fig 10(a) and 10(c)), but still weaker than *Starling*(*G*, *τ* = 0.25), whereas *Infomap* can never succeed, because its (*i*) behavior.

**To sum up**: If we know the good number of groups of vertices *κ* in advance then we can use *SGC*. If we do not know it, then we can use *Infomap* on the one hand with *Starling* on the other hand wich are complementary:

*Infomap* tend to favor *Recall* with good *Fscore* and is able to identify the absence of strong structures;
*Starling*^*τ*=0.25 *by default*^ tends to favor *Precision* with good *Fscore* and is able to identify the presence of weak structures. Then if we want to promote *Recall* with a smaller number of larger Modules, we can decrease *τ*, and if we want to promote *Precision* with a greater number of smaller Modules, we can increase *τ*.

**Our follow-up work**: We will focus on the role on the ouputs of *Starling*, played by the length of the Random walks in computing *Confluence*, as well as the development of a Clustering method based on *Confluence* able to detect Clustering in Graphs accounting for edge directions and edge weights, its returns communities possibly overlapping.

## Supporting information

S1 Fig (TIF)
