# Supplementary figures and images for: Starling: Introducing a mesoscopic scale with Confluence for Graph Clustering
Source: PLoS One. 2023 Aug 24;18(8):e0290090. doi: 10.1371/journal.pone.0290090 (PMC10449208; doi:10.1371/journal.pone.0290090)

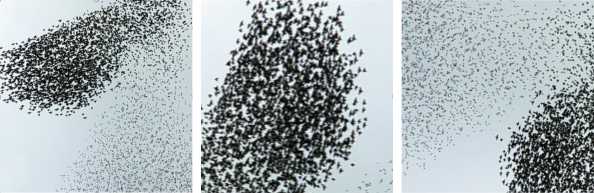

Supplement: S1 Fig — (TIF) [file pone.0290090.s001.tif]
